# Spin Polarization and Flat Bands in Eu-Doped Nanoporous and Twisted Bilayer Graphenes

**DOI:** 10.3390/mi14101889

**Published:** 2023-09-30

**Authors:** Iu. A. Melchakova, G. T. Oyeniyi, S. P. Polyutov, P. V. Avramov

**Affiliations:** 1 School of Physics and Engineering, ITMO University, 197101 St. Petersburg, Russia; iuliia.melchakova@metalab.ifmo.ru; 2 Department of Chemistry, Kyungpook National University, Daegu 41566, Republic of Korea; oyeniyigbenga12@gmail.com; 3 International Research Center of Spectroscopy and Quantum Chemistry (IRC SQC), Siberian Federal University, Svobodniy pr. 79/10, 600041 Krasnoyarsk, Russia; polyutov@mail.ru

**Keywords:** twisted bilayer graphene, bilayer graphene, nanoporous bilayer graphene, flat bands, spin polarization, DFT

## Abstract

Advanced two-dimensional spin-polarized heterostructures based on twisted (TBG) and nanoporous (NPBG) bilayer graphenes doped with Eu ions were theoretically proposed and studied using Periodic Boundary Conditions Density Functional theory electronic structure calculations. The significant polarization of the electronic states at the Fermi level was discovered for both Eu/NPBG(AA) and Eu/TBG lattices. *Eu* ions’ chemi- and physisorption to both graphenes may lead to structural deformations, drop of symmetry of low-dimensional lattices, interlayer fusion, and mutual slides of TBG graphene fragments. The frontier bands in the valence region at the vicinity of the Fermi level of both spin-polarized 2D Eu/NPBG(AA) and Eu/TBG lattices clearly demonstrate flat dispersion laws caused by localized electronic states formed by TBG Moiré patterns, which could lead to strong electron correlations and the formation of exotic quantum phases.

## 1. Introduction

Quantum phases [1,2,3] are strongly correlated electronic states that can be formed under specific conditions near 4 K temperature. Even at such low temperatures, any quantum electronic subsystem may be influenced by quantum fluctuations caused by a sudden change in occupation numbers or in the symmetry of electronic states. As a physical parameter is varied, quantum fluctuations can cause a sudden phase transition, like superconductor-Mott insulator transition, which separates two quantum phases that have different electronic symmetries with entirely different transport properties. Quantum phase transitions are sudden and fundamental rearrangements of strongly correlated electronic systems as they evolve from well-defined excitations in Mott insulators to a completely different set of excitations in superconducting phases of high-temperature superconductors [4,5].

The connection between localized electronic states and electronic correlations is a fundamental concept in condensed matter physics and materials science [4,5,6,7,8,9,10,11]. Electron confinement caused by the localization of some electronic states leads to electron–electron correlations with the formation of either superconducting Cooper electron pairs or Mott insulators, in which electron–electron repulsion dominates. The close proximity of the localized non-overlapping electronic states leads to pronounced strong electron correlations, which determine the main physical properties of crystalline lattices, which often exhibit unique and exotic electronic properties, such as high-temperature superconductivity, metal-insulator transitions, and magnetism. For such atomic crystalline lattices, traditional band theory, which assumes weak correlations, breaks down, and more advanced theoretical approaches, like the Hubbard, Anderson, t-J, etc., models, are used to describe the electronic behavior. Localized electronic states, especially when they are strongly localized, can lead to enhanced electronic correlations due to the close proximity and enhanced Coulomb repulsion of the electronic subsystems.

In crystalline lattices which are characterized by flat electronic bands, the electronic states are typically highly localized in real space [12]. This means that electrons within the flat bands tend to be concentrated in specific regions or sites within the crystal lattice. This enhanced electron–electron interaction is a hallmark of correlated electron systems. The strong electron–electron interactions within flat bands could lead to pronounced correlation effects and play a crucial role in determining the material’s properties. For example, the presence of flat bands can enhance the likelihood of electron pairing, which can be a key factor in the emergence of superconductivity or other exotic electronic states.

The unique physical properties of 2D bigraphenes (BGs) [13] make them promising 2D materials for advanced photo- [14], spin [15], and quantum applications [16]. For instance, BG-based superlattices, such as nanoporous bigraphene (NPBG) or twisted bilayer graphenes (TBGs), were introduced and studied theoretically [17,18] and experimentally [19,20] with multiple evidences of remarkable quantum and spin properties.

At particular magic angles with the largest one of around 1.05° [21], the Moiré patterns created by the twisted layers cause the electronic bands of the material to become nearly flat near the Fermi level and cause strong electron–electron interactions and correlations. At magic angles TBGs exhibit challenging strongly correlated [21,22,23,24], Mott insulating [25], superconducting [26], ferromagnetic [27,28,29], and topological [21,30] phases caused by the orbital motion of the valence electrons rather than their spins [31]. Strongly correlated systems [32] may lead to superconductivity and long-range quantum entanglement. Flat valence bands in the vicinity of the Fermi level may determine the main physical properties of low-dimensional lattices since the energy range of the bands is so narrow that the Coulomb interactions dominate over kinetic energy, putting these materials in a strongly correlated regime.

Twisted bilayer graphene at magic angles and at higher temperatures T_c_ is a strongly correlated Mott insulator [33], which is characterized by a non-conducting ground state produced by strong repulsive interactions between the electrons. During the quantum phase transition at T_c_~0 K [1], the insulator turns into a superconductor when a small number of charge carriers are added to the bigraphene lattice. The stacking of graphene sheets allows access to a new family of materials with electronic behavior that is exquisitely sensitive to the atomic alignment between the layers, which affects interlayer electron motion. At very low rotation angles, Moiré patterns are produced by the misaligned lattices with dramatically enlarged unit cells and with completely reconstructed electronic states, which leads to strong correlation coupling between the electrons localized at different graphene layers. New narrow bands emerge at rotation angles below 1.05° when TBG is close to charge neutrality. The electrons in these narrow bands in the vicinity of the Fermi level are found mainly in the regions of the Moiré pattern, in which the atoms are stacked directly above each other, and TBG can be thought of as a triangular lattice of weakly coupled quantum dots with a residual tunnelling of electrons between them. The adding of charge carriers to twisted bilayer graphene leads to a Berezinskii–Kosterlitz–Thouless phase transition with T_c_ around 1.7 K with the formation of 2D superconducting quantum phase.

Recently, it was shown that superlattices of potential bumps driven by folded holes in nanoporous bigraphene may induce visible spin polarization by breaking the balance between two graphene sublattices, thereby creating unpaired spins [34]. Dai et al., discovered that the graphene sheet’s magnetism only appears in vacancies with unpaired electrons, opening the door to manipulate the magnetism in these two-dimensional materials [35]. As a result, due to the presence of vacancy-related defects in graphene’s lattice, experimental studies were able to discover magnetic moments ranging from 1.0 to 1.5 μ [36,37,38]. It was shown that NPBG may exhibit distinct spin polarization due to intense local electric fields generated by doping graphene lattice by spin non-polarized *sp*-metals, such as Li, Ca, and Al [17]. It could be a significant step forward since any mechanism that allows for the manipulation of magnetism at the nanoscale is essential for designing novel devices for spin-related applications.

The spin polarization of graphene-based low-dimensional materials can be achieved by the creation of either complex heterostructures or defect lattices based on transition metal dichalcogenides/graphene [39] heterostructures, adatom lattices [40], vacancies [38], or zig-zag edges [41]. Spin polarization is caused by the breakdown of lattice inversion symmetry and the asymmetry of spin-up and spin-down Density of Electronic States (DOS) when one spin state could become more populated than the other [38]. In particular, spin polarization could be induced by the application of a magnetic field [42], which could affect spin transport in graphene as well as the use of rare-earth metals [43].

High magnetoresistance in graphene was discovered in graphene coordinated to EuO and EuS surfaces [44]. It was suggested that the EuO/graphene heterostructures composed of a sufficiently wide and short graphene sheet and gated EuO strips would pave the way to design advanced spintronic devices [44]. Furthermore, it was suggested that coupling graphene to the model magnetic EuS insulator produces a significant (>14 T) magnetic exchange field (MEF) with the potential to reach hundreds of tesla, resulting in an orders-of-magnitude enhancement of the spin signal originating from the Zeeman spin Hall effect [45].

Experimental data demonstrated that both EuS/graphene [45] and EuO/graphene [46] heterostructures generate spin polarization that can function as 2D spin logic and memory devices that would be useful in various advanced quantum applications. A possible synthesis for Eu/bilayer graphene could be achieved by europium deposition on single-layer graphene transferred to a common SiO_2_/Si substrate. There are two main options for its synthesis: the intercalation of Eu under the graphene sheet or the adsorption of Eu on top of the material [46,47,48,49]. It is well known that Eu intercalation exhibits intricate structural patterns with stripes, compact islands, and channels [50]. It is possible to create ordered superstructures of intercalated Eu under the conditions of Eu distillation, where a high-temperature regime aids in the evaporation of the excess Eu [51,52]. At a high-temperature regime, however, one cannot preclude the formation of Eu nanoclusters between the substrate and the graphene [53]. As a result, the adsorption of Eu on graphene is preferable, which is predicted to proceed under milder conditions. However, this synthetic approach is more difficult since it necessitates that the amount of deposited Eu fit the EuC_6_ stoichiometry for single-layer graphene.

The main goal of this investigation is theoretical design and the study of complex heterostructures based on nanoporous and twisted bigraphenes with either adsorbed or intercalated Europium ions using Density Functional Theory Periodic Boundary Conditions electronic structure calculations. It was shown that Eu-doping causes spin polarization with a different spatial localization of α and β spin states and fundamental alteration of their band structures with the formation of flat bands in the vicinity of the Fermi level, which may promote strong electronic correlations with the formation of quantum phases, which are promising for advanced spin- and quantum-related applications.

## 2. Computational Details

The electronic structure calculations of low-dimensional crystalline lattices were performed using the Vienna Ab-initio Simulation Package (VASP) [54,55,56] within Density Functional Theory (DFT) [57,58] and Periodic Boundary Conditions (PBC). Plane-wave basis set coupled with the projector augmented wave (PAW) method [59,60], GGA-PBE [61] functional, and Grimme D3 correction [62] for Van der Waals interaction were used in the study. Taking into account the correlation effects, the simplified form of Hubbard U correction proposed by Dudarev et al. [63,64] was implemented with U = 7.5 eV and J = 0.6 eV [65]. Mönkhorst-Pack Brillouin zone *k*-point sampling was implemented, and the *k*-point mesh contained 3 × 3 × 1 *k*-points along with *a*, *b*, and *c* directions, respectively, being used for carbon-based media optimization. For all elements involved in the electronic structure calculations, PAW potentials were used. For carbon, 4 outer electrons, and for Eu, 8 outer electrons, were treated as valence electrons. For Eu, semi-core *f*-electrons were treated as core states despite being higher in energy than other valence states. For the density of states calculations, the 6 × 6 × 1 *k*-point mesh was used. A vacuum interval of 20 Å was set normal to the plane to avoid artificial interactions between adjacent unit cell images. In all calculations, the cut-off energy was equal to 600 eV. During the optimization procedure, the maximum force acting on atoms less than 0.001 eV/Å was used as a stopping criterion for structural minimization.

## 3. Results and Discussion

### 3.1. Crystalline Lattices of Proposed Heterostructures

**Figure 1:**
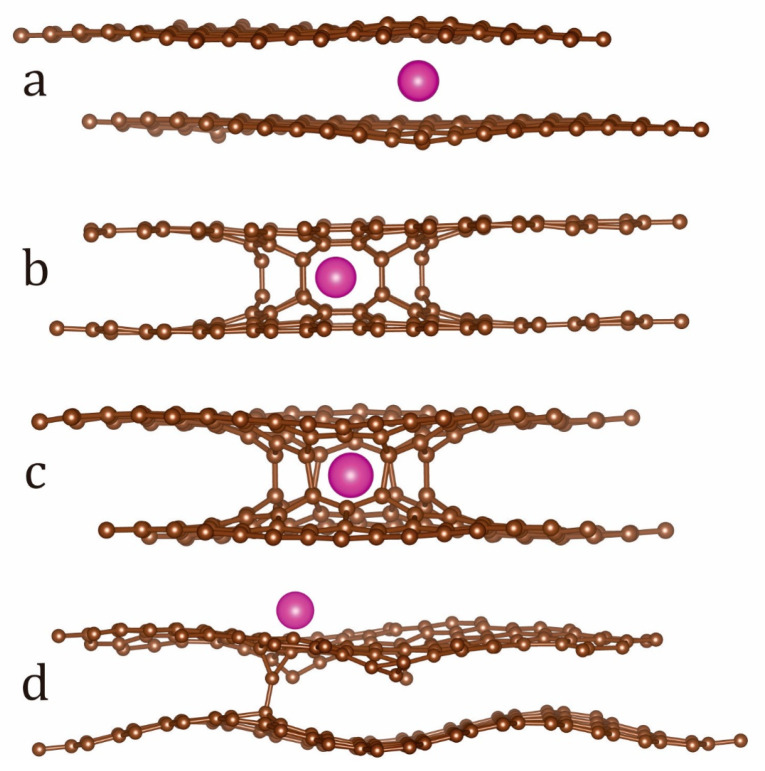
The atomic lattices of bigraphene-based heterostructures, namely Eu/BG (**a**), Eu/NPBG(AA) (**b**), Eu/NPBG(AB) (**c**), and Eu/TBG, with a (**d**) side view. Eu atoms are depicted in purple; carbon atoms are depicted in brown.

Initial atomistic models of the heterostructures were developed using (i) AA and AB bigraphenes [5] with either adsorbed or intercalated Eu ions; (ii) AA and AB NPBG [17], and (iii) twisted bilayer graphene had a twisting angle equal to 21.8° (TBG-21; but for the simplicity, it will be referred to simply as TBG, Figure 1). For the sake of comparison, the structure of pristine AA and AB bigraphenes with 0 twisting angle and both Eu-doped/intercalated AA and AB bigraphenes with 0 twisting angle were calculated as well. The Eu atom was placed inside the pore of Eu/NPBG, atop of Eu/TBG, and between carbon-based fragments (Eu/BG) with the consequent structural optimizations. The structural parameters (number of atoms per unit cell, magnetic moments, and unit cell parameters) are displayed in Table 1. The initial symmetry of carbon-based BG, TBG, and NPBG precursors was found to be hexagonal with *a* = *b* and *γ* = 120°, but during structural optimization, a drop of symmetry had been found for some of them. The unit cells of all heterostructures are presented in the Supplementary Materials.

For the sake of comparison, the optimization of the 2D atomic lattices of parent TBG-21 and both AA/NPBG and AB/NPBG was performed using the same PBC GGA-PBE + U PAW D3 approach. Structural optimization revealed small effects of DFT + U corrections on structure and electronic properties of all 2D lattices. The Parent TBG-21 lattice suffered small structural distortions constituting graphene fragments, which drastically increased with the Ei adsorption.

**Figure 2:**
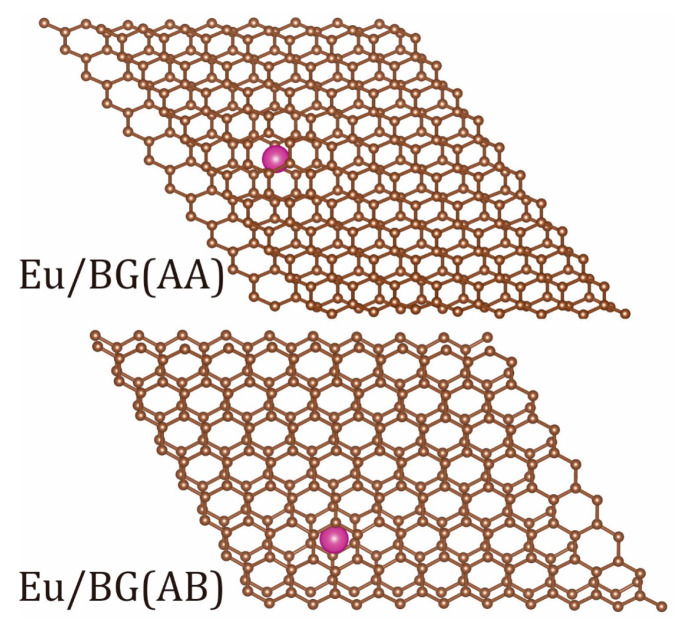
The top view of atomic lattices of Eu/BG (AA) (**top**) and Eu/BG (AB) (**bottom**) heterostructures. Eu atoms are depicted in purple, carbon atoms are depicted in brown.

The PBE PBC atomic structure optimization of Eu/BG-AA and Eu/BG-AB experienced drastic cell distortion. In both cases, the lattice symmetry dropped from hexagonal to monoclinic with some slides of graphene fragments (Figure 2) along the *xy* direction. In both BG (AA) and BG (AB) cases the Eu interlayer led to the bending of the graphene fragments induced by repulsion with Eu ions.

Topological stability of complex low-dimensial crystalline lattices with multiple non-equivalent sublattices is a key issue for the design and study of 2D periodic atomic lattices from both from theoretical [56] and experimental [66] points of view. In contrast to perfectly planar regular hexagonal/triangular symmetry 2D graphene, *h*-BN, *g*-C_3_N_4_, *g*-C_4_N_3_, MoS_2_, and MoSe_2_ lattices [67,68,69] may form considerably complex shapes, like rolls, nanotubes, aperiodic flakes and structure waves, caused by the slight or moderate mutual structural mismatch of structural units, like pentagons, hexagons, heptagons, etc., which constitute complex low-dimensional periodic atomic lattices. Following Topology Conservation Theorem [70] the lattices may release the excess of artificial mechanical stress caused by artificial symmetrical constrains of a perfectly planar lattice implemented by inappropriate application of Periodic Boundary Conditions through bending with out-of-plane expansion.

**Figure 3:**
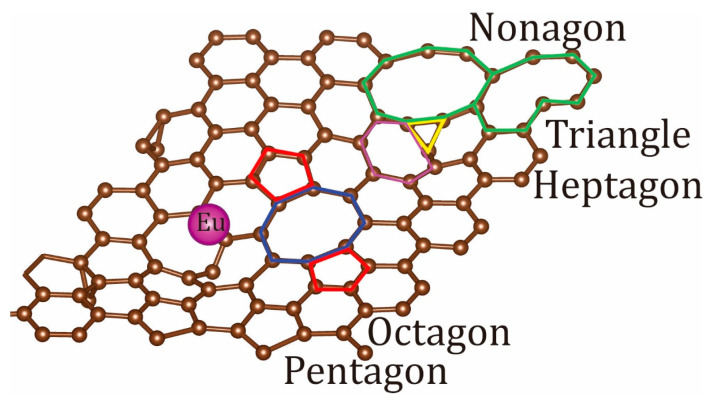
The structure and symmetry of Eu/TBG unit cell. Eu atom is depicted in purple, carbon atoms are depicted in brown, the triangular fragment is depicted in yellow, pentagonal fragments are depicted in red, the heptagonal fragment is depicted in purple, the octagonal fragment is depicted in blue, and the nanogonal fragment is depicted in green.

The lattices of the graphene fragments of Eu/TBG demonstrated significant reconstruction during structural optimization, with the formation of corrugated carbon lattices formed by triangles, pentagons, hexagons, heptagons, octagons, and even nonagons (Figure 3). In particular, Eu/TBG demonstrated a non-planar wave-shaped lattice together with interlayer stitching through covalent carbon–carbon bonds, which prevents the rolling of the heterostructure [70]. The interaction between TBG and Eu-induced structural recombination combined with the out-of-plane distortions is responsible for the reduction in mechanical stress and the relaxation of the fragments of the entire interface.

### 3.2. Electronic Structure and Spin States of Eu-Doped Bigraphene-Based Heterostructures

**Figure 4:**
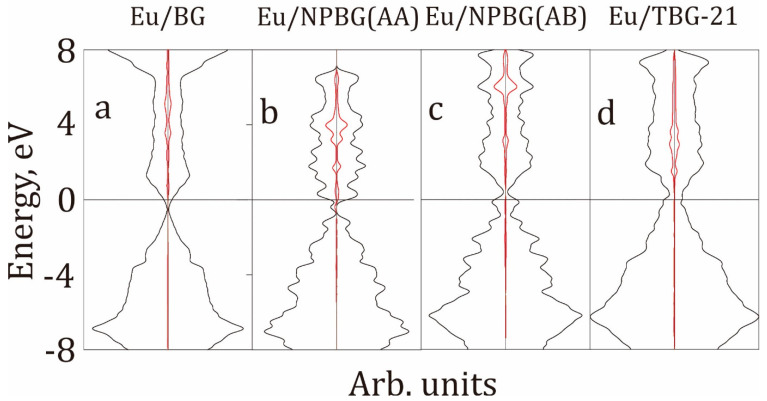
(**a**) Total (TDOS) and partial (PDOS) density of states of Eu coordinated to bigraphene (Eu/BG), (**b**) AA nanoporous bigraphene (Eu/NPBG (AA), (**c**) AB nanoporous bigraphene (Eu/NPBG (AB), and (**d**) twisted bilayer graphene 21 (AA nanoporous bigraphene, Eu/TBG). TDOSes are depicted in black, and Eu PDOSes are depicted in red.

The analysis of Total (TDOS) and Partial (PDOS) Density of Electronic States for Eu/BG (Figure 4a) unequivocaly demonstrates its spin non-polarized nature. The valence electronic bands (VB) are formed by BG fragments contributions, while conduction bands (CB) involve a partial contribution of Eu adatom. The band structure (Figure S1) demonstrates the shift of the graphene Dirac cone by 0.4 eV down to the VB region. The DOS analysis of Eu/NPBG (AA) (Figure 4b) demonstrates the presence of distinctive spin polarization at the Fermi level with the absence of spin polarization in the [−8, −0.5] eV and [0.5, 8] eV energy regions. VB are formed by the NPBG (AA) fragment, while CB formation involves the partial contribution of Eu adatom together with the contribution of the NPBG (AA) fragment. The analysis of graphene and Eu partial density of states of Eu/NPBG (AB) (Figure 4c) demonstrates its spin non-polarized nature as well with CB mostly formed by the NPBG (AB) fragment with minor contributions of Eu states, and VB formed just by NPBG (AB). The DOS analysis of Eu/TBG (Figure 4d) demonstrates distinctive spin polarization in the [−0.5, 0.5] eV region, with VB formed by PDOS TBG fragment, and CB formed by PDOSes of TBG and Eu states.

To study the impact of DFT + U corrections, the U_f_ = 7.5 eV and J_f_ = 0.6 eV parameters [65] were chosen to treat strong electronic correlations defined by the presence of Eu *f*-electrons. The results of DOS and band structure calculations (Figures S3 and S4) demonstrated the absence of any impact of Hubbard correction at electron density at the Fermi level due to the small Eu contribution, i.e., the electron density at the Fermi level is mostly formed by carbon-based fragments consisting of only *s*- and *p*-shell-occupied electrons.

**Figure 5:**
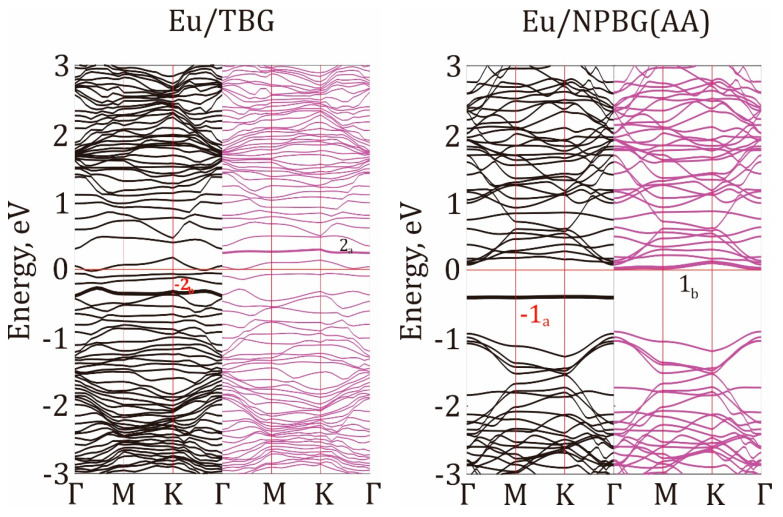
The band structure of Eu/TBG (**left panel**) and Eu/NPBG (AA) (**right panel**) heterostructures. Spin-up (α) bands are depicted in black, and spin-down (β) bands are depicted in purple.

The band structure of spin-polarized Eu/NPBG (AA) and Eu/TBG heterostructures are presented in Figure 5. Both lattices can be assigned as direct flat band semiconductors because the curvature of the valence bands is smaller than the accuracy of both hybride PBE0 and GGA-PBE functionals, which is equal to 2–3 kcal/mol [71,72]. Band structure analysis proved the spin polarization of the heterostructures only in the vicinity of the Fermi levels, as it was found during DOS analysis (see above). Spin polarization could be seen in the [−0.5, 0.5] eV region for both Eu/NPBG and Eu/TBG. The symmetry of the bands and its comparison between α and β states demonstrate significantly different band dispersion for the spin-up and spin-down electrons. The shape of the second conduction band for the α spin state (−2_a_) is similar to the shape of the second valence band for the β spin state (2_b_). This fact can indicate the correspondence of the −2_a_ band to the 2_b_ band, induced by the Eu coordination atop TBG since the DOS at the Fermi level is fully determined by the TBG fragment (Figure 4d). Tuning the electronic structure of graphene to control spectroscopic properties was discussed as well in Refs. [73,74,75].

The band shift could be identified in the case of the Eu/NPBG (AA) interface as well. The first flat valence band of the α spin state (−1_a_) looks fairly similar to the first flat conduction band of the β spin state (1_b_), so the −1_a_ band-to-1_b_ band correspondence can occur. The flat nature of the band dispersion law for boundary CB and VB demonstrates their localized nature and the absence of chemical interactions between the states. In case of flat bands, the electronic transitions between VB and CB would not experience phonon relaxation, which could enhance the transition performance.

**Figure 6:**
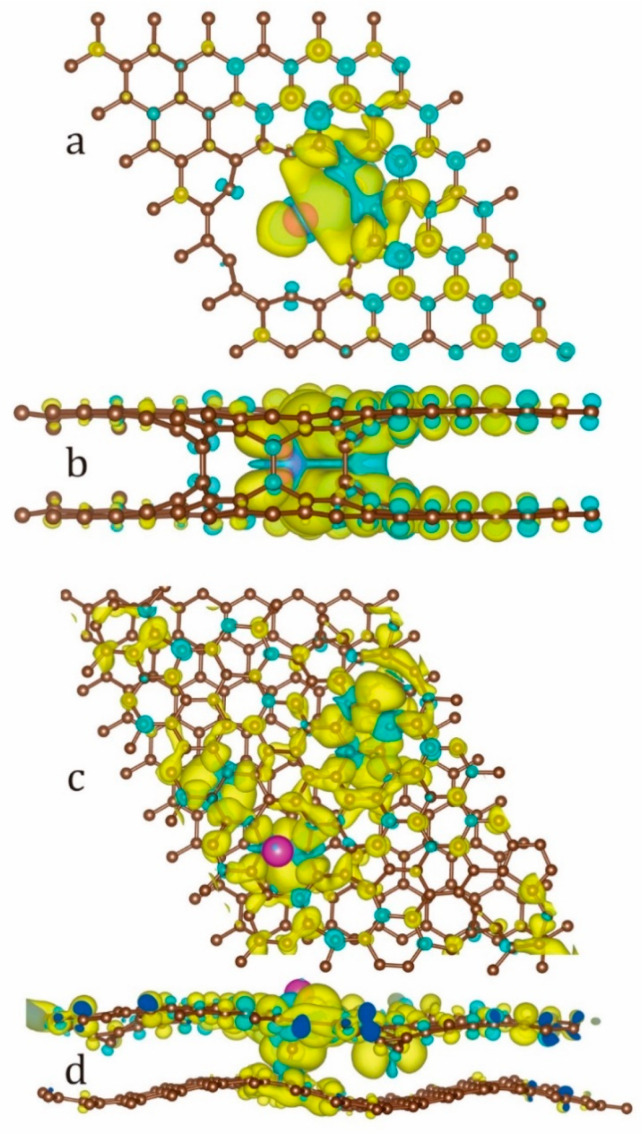
(**a**,**b**) Top and side views of spin density distribution for Eu/NPBG (AA), respectively. (**c**,**d**) Top and side views of spin density distribution for Eu/TBG. Spin-up (α) density is depicted in yellow, and spin-down (β) density is depicted in cyan. Carbon atoms are depicted in brown and Eu atom is depicted in purple.

The spin density distribution for Eu/NPBG (AA) and Eu/TBG are presented in Figure 6. The different spatial localization of α and β spin states is the direct evidence of the spin-polarized nature of the heterostructures. Both α and β spin densities are localized at both the Eu atom and TBG or NPBG AA fragments. In the case of Eu/NPBG (AA), the spin density is spreading to the NPBG pore, inducing the electronic redistribution of the NPBG fragment (yellow for the α channel and cyan for the β one).

In particular, for Eu/TBG, the spin density is fully localized at the TBG fragment (Figure 6c,d). Neither α (yellow) nor β (cyan) spin densities could be found at the Eu atom, while in the case of the TBG fragment, spin density was distributed for the entire unit cell. The length of C-Eu bonds (2.65 Å for Eu/NPBG (AA) and 2.64 Å for the Eu/TBG) directly indicates the absence of strong C-Eu covalent bonding, while the spin polarization of the fragments could be promoted simply by the exchange interactions between the states.

## 4. Conclusions

In this study, the key Eu-doped low-dimensional 21.8° twisted bilayer graphene and both AA and AB nanoporous bigraphenes were theoretically proposed and studied using the PW PBC DFT approach. Based on electronic structure calculations, it was shown that Eu coordination on top of both the bigraphene and twisted bilayer graphene induces the glide of graphene fragments and drastic structural deformation for Eu/TBG. In accordance with the Topology Conservation Theorem, corrugated TBG graphene fragments form structural waves coupled with interlayer graphene fusion, which would stabilize the local structure and the long-order topology of 2D graphene lattice. The electronic structure calculations revealed that both Eu/NPBG (AA) and Eu/TBG are spin-polarized direct flat band semiconductors with a spin density mostly localized at graphene fragments. The localization of uncompensated spins highlights the leading role of carbon fragments in the spin polarization of the heterostructures. Heterostructure DOSes clearly demonstrate the similar distribution of electronic density of both valence and conductivity bands in the vicinity of the Fermi levels. The total density of states of the valence bands is mostly formed by carbon contributions, while conduction bands have some contributions to the Eu partial electronic states. The distinctive spin polarization and flat bands of the proposed graphene-based low-dimensional quantum materials may promote advanced spin- and quantum-related applications.

## Figures and Tables

**Table 1:** The structural parameters (Å) and magnetic moments (µ_B_) of bigraphene-based heterostructures doped with Eu ions.

	Number of Atoms Per Cell, N_at_	Magnetic Moment Per Cell, µ_B_	Cell Parameter *a*, Å	Cell Parameter *b*, Å
Eu/BG (AA)	197	0.00	17.26	17.26
Eu/BG (AB)	197	0.00	17.25	17.26
Eu/TBG	195	4.14	16.93	16.90
Eu/NPBG (AA)	133	0.74	14.74	14.74
Eu/NPBG (AB)	137	0.00	14.72	14.72

## Data Availability

All data available in the text of the paper, SI Section and on request to the authors.

## Supplementary Materials

The following supporting information can be downloaded at: https://www.mdpi.com/article/10.3390/mi14101889/s1. Figure S1. The band structure of Eu/BG heterostructure. Spin-up (α) bands are depicted in black, and spin-down (β) bands are depicted in purple. Figure S2. The band structure of Eu/NPBG heterostructures. Spin-up (α) bands are depicted in black, and spin-down (β) bands are depicted in purple. Figure S3. The band structure of Eu/TBG heterostructures calculated at GGA PBE (left panel) and GGA PBE+U (right panel) levels of theory. Spin-up (α) bands are depicted in black, and spin-down (β) bands are depicted in purple. Figure S4. Total density of states (TDOS) of Eu/TBG calculated using DFT (black curve), DFT+U with f-electons treated by Dudarev method (green curve), and DFT+U with d-electons treated by Dudarev method (purple curve). Crystal data are provided for: Eu/BG (AA), Eu/BG (AB), Eu/NPBG (AA), Eu/NPBG (AB), Eu/TBG.
