# Supplementary material for: Spin Polarization and Flat Bands in Eu-Doped Nanoporous and Twisted Bilayer Graphenes
Source: Micromachines (Basel). 2023 Sep 30;14(10):1889. doi: 10.3390/mi14101889 (PMC10609095; doi:10.3390/mi14101889)
Supplement: Supplementary file 1 [file micromachines-14-01889-s001.zip › micromachines-2630891-supplementary.pdf]

Spin Polarization and Flat Bands in Eu-doped nanoporous and twisted bilayer graphenes

Iu. A. Melchakova, G. T. Oyeniyi, S. P. Polyutov and P. V. Avramov

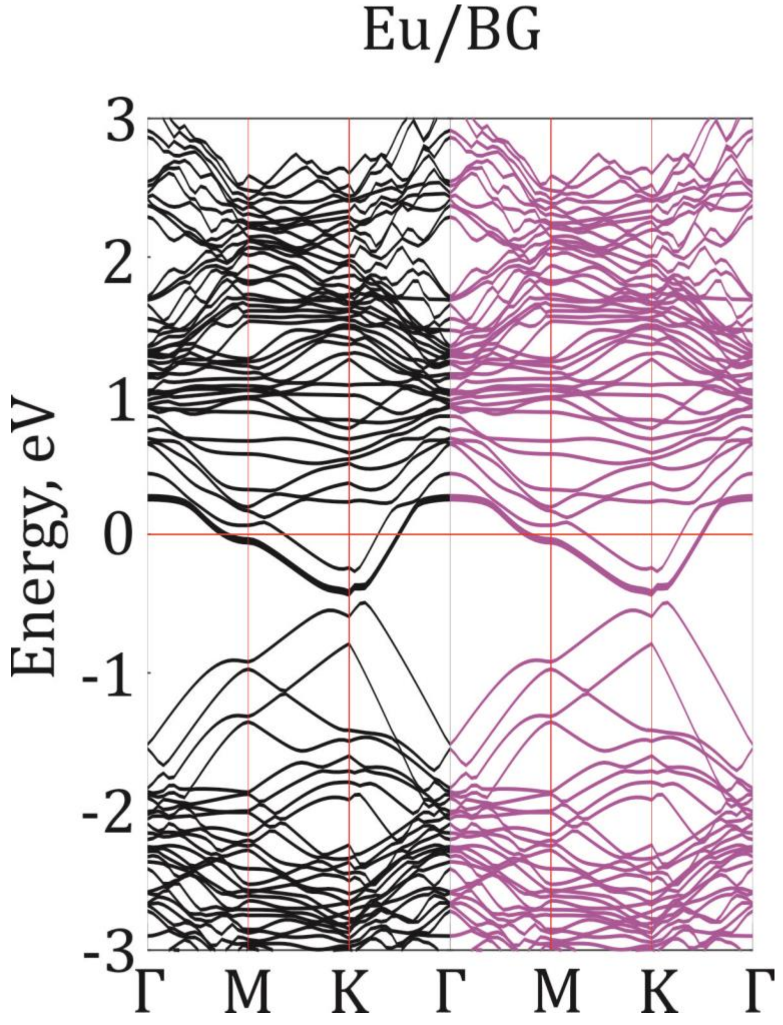

Figure S1. The band structure of Eu/BG heterostructure. Spin-up (α) bands are depicted in black, and spin-down (β) bands are depicted in purple

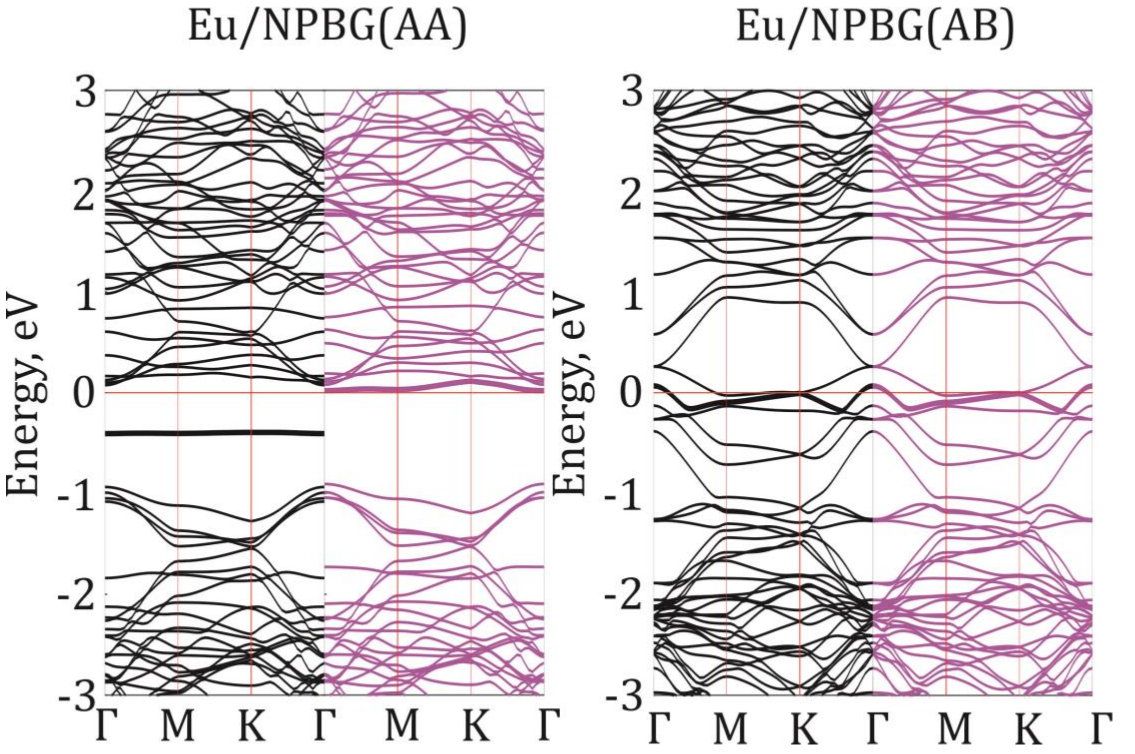

Figure S2. The band structure of Eu/NPBG heterostructures. Spin-up (α) bands are depicted in black, and spin-down (β) bands are depicted in purple

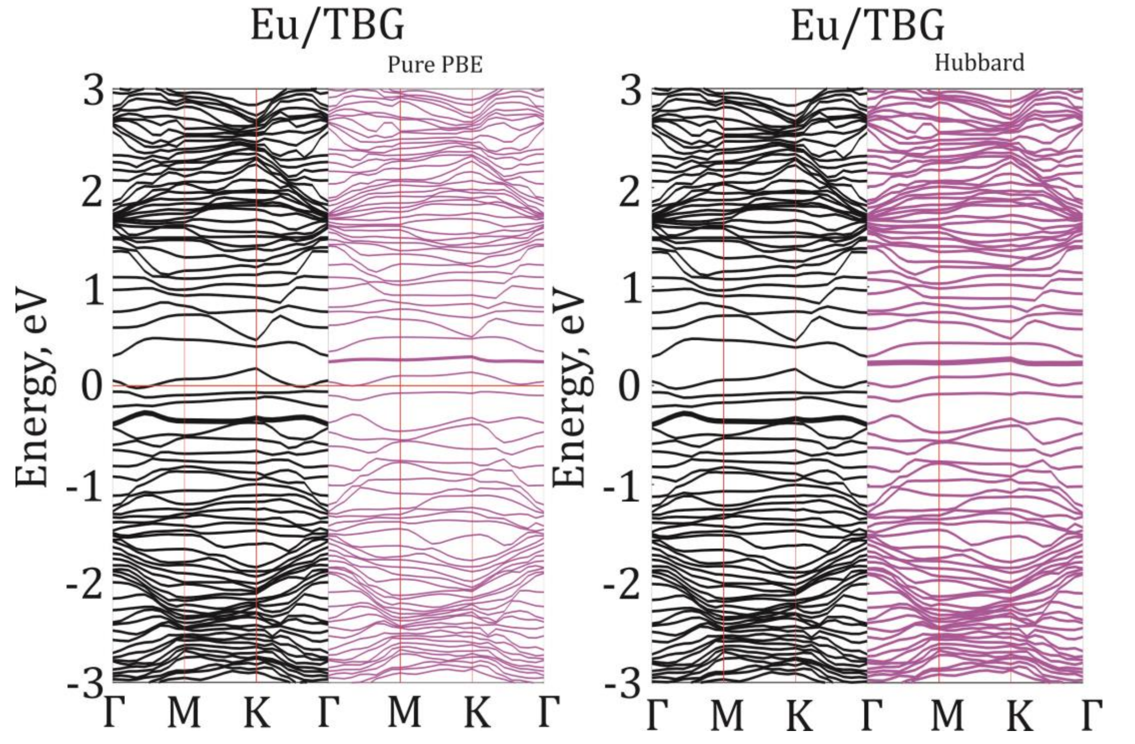

Figure S3. The band structure of Eu/TBG heterostructures calculated at GGA PBE (left panel) and GGA PBE+U (right panel) levels of theory. Spin-up (α) bands are depicted in black, and spin-down (β) bands are depicted in purple

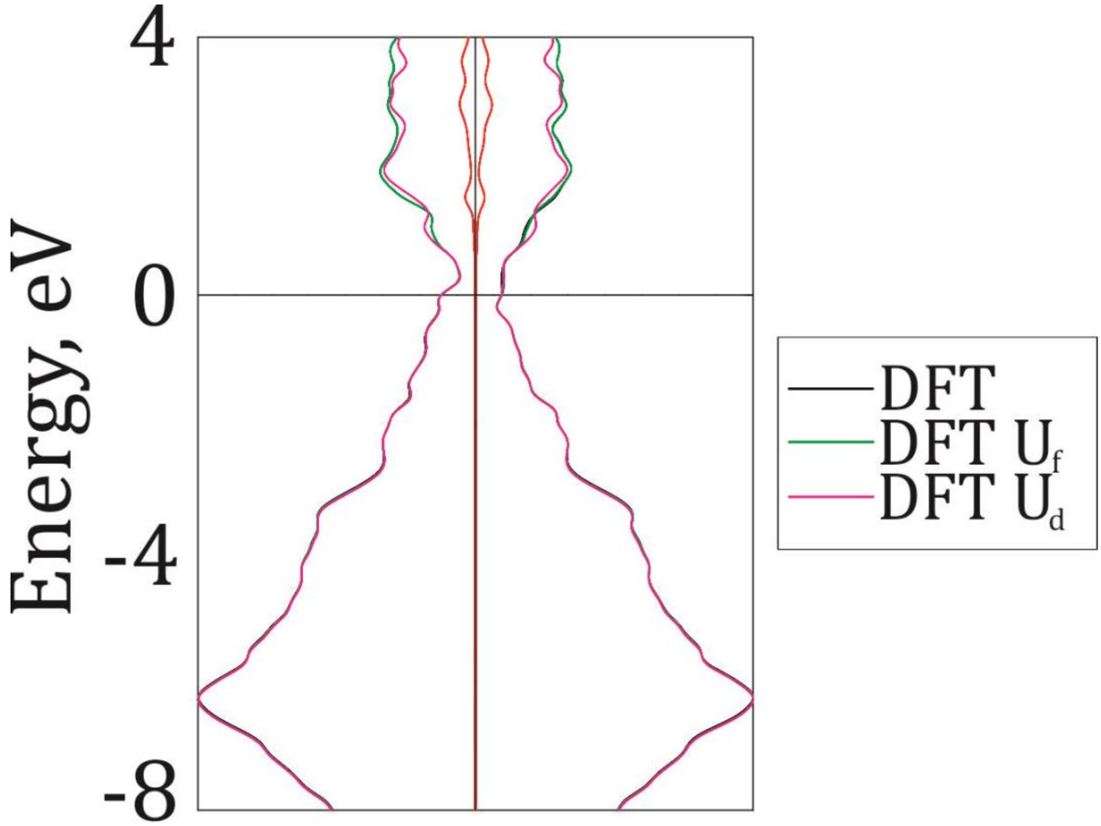

Figure S4. Total density of states (TDOS) of Eu/TBG calculated using DFT (black curve), DFT+U with *f*-electons treated by Dudarev method (green curve), and DFT+U with *d*-electons treated by Dudarev method (purple curve)

1. Eu-BG(AA).cif

```
#======================================================================
# CRYSTAL DATA
#----------------------------------------------------------------------
data_VESTA_phase_1

_chemical_name_common                  'Eu/BG (AA) '
_cell_length_a                         17.259382
_cell_length_b                         17.257092
_cell_length_c                         31.364382
_cell_angle_alpha                      93.774475
_cell_angle_beta                       101.447136
_cell_angle_gamma                      120.034439
_cell_volume                           7774.917192
_space_group_name_H-M_alt              'P 1'
_space_group_IT_number                 1

loop_
_space_group_symop_operation_xyz
   'x, y, z'

loop_
   _atom_site_label
   _atom_site_occupancy
   _atom_site_fract_x
```

_atom_site_fract_y

_atom_site_fract_z

_atom_site_adp_type

_atom_site_U_iso_or_equiv

_atom_site_type_symbol

C1 1.0 0.613777 0.734445 0.192903 Uiso ? C

C2 1.0 -0.119902 -0.095197 0.075494 Uiso ? C

C3 1.0 -0.338060 -0.169905 0.193797 Uiso ? C

C4 1.0 -0.072533 -0.000185 0.074961 Uiso ? C

C5 1.0 -0.243571 0.734237 0.192274 Uiso ? C

C6 1.0 0.019393 0.902201 0.069917 Uiso ? C

C7 1.0 -0.195148 -0.169931 0.193597 Uiso ? C

C8 1.0 0.065484 -0.003443 0.067028 Uiso ? C

C9 1.0 -0.099095 0.735417 0.195086 Uiso ? C

C10 1.0 0.160002 0.900173 0.065277 Uiso ? C

C11 1.0 -0.050340 -0.168499 0.197124 Uiso ? C

C12 1.0 0.204025 -0.007264 0.058541 Uiso ? C

C13 1.0 0.045937 0.737044 0.199199 Uiso ? C

C14 1.0 0.306485 0.902672 0.071150 Uiso ? C

C15 1.0 0.095148 -0.166640 0.202400 Uiso ? C

C16 1.0 0.353386 -0.002460 0.069606 Uiso ? C

C17 1.0 0.189561 0.737556 0.200582 Uiso ? C

C18 1.0 0.452368 -0.094715 0.076855 Uiso ? C

C19 1.0 0.238928 -0.166146 0.203720 Uiso ? C

C20 1.0 0.500140 0.000660 0.077321 Uiso ? C

C21 1.0 0.331321 0.736808 0.198669 Uiso ? C

C22	1.0	0.596275	-0.093902	0.078806	Uiso	? C
C23	1.0	0.379984	-0.167259	0.200458	Uiso	? C
C24	1.0	0.644346	0.001661	0.079668	Uiso	? C
C25	1.0	0.472411	0.735560	0.195612	Uiso	? C
C26	1.0	0.738840	-0.094100	0.078207	Uiso	? C
C27	1.0	0.520580	-0.168792	0.196550	Uiso	? C
C28	1.0	0.786741	0.001311	0.078691	Uiso	? C
C29	1.0	0.615298	-0.121572	0.195586	Uiso	? C
C30	1.0	-0.118703	0.048471	0.077511	Uiso	? C
C31	1.0	-0.336342	-0.025838	0.196904	Uiso	? C
C32	1.0	-0.070583	0.144064	0.078479	Uiso	? C
C33	1.0	-0.242615	-0.122141	0.194076	Uiso	? C
C34	1.0	0.020679	0.046163	0.071646	Uiso	? C
C35	1.0	-0.194074	-0.026273	0.195740	Uiso	? C
C36	1.0	0.069031	0.141965	0.073060	Uiso	? C
C37	1.0	-0.098581	-0.121338	0.196051	Uiso	? C
C38	1.0	0.157388	0.041950	0.061250	Uiso	? C
C39	1.0	-0.049765	-0.025229	0.198322	Uiso	? C
C40	1.0	0.208502	0.139620	0.066984	Uiso	? C
C41	1.0	0.047140	-0.119268	0.201697	Uiso	? C
C42	1.0	0.302869	0.042940	0.063943	Uiso	? C
C43	1.0	0.096576	-0.022596	0.205521	Uiso	? C
C44	1.0	0.353240	0.140435	0.069318	Uiso	? C
C45	1.0	0.192404	-0.117755	0.206143	Uiso	? C
C46	1.0	0.451173	0.047296	0.074771	Uiso	? C
C47	1.0	0.243042	-0.020585	0.211944	Uiso	? C

C48	1.0	0.499972	0.143342	0.076938	Uiso	?	C
C49	1.0	0.334353	-0.118420	0.203988	Uiso	?	C
C50	1.0	0.596646	0.049235	0.079655	Uiso	?	C
C51	1.0	0.383956	-0.021928	0.207135	Uiso	?	C
C52	1.0	0.645137	0.145079	0.081264	Uiso	?	C
C53	1.0	0.474533	-0.120135	0.199254	Uiso	?	C
C54	1.0	0.739824	0.049460	0.080040	Uiso	?	C
C55	1.0	0.523024	-0.024301	0.200769	Uiso	?	C
C56	1.0	0.788144	0.145160	0.081337	Uiso	?	C
C57	1.0	0.617274	0.022654	0.199076	Uiso	?	C
C58	1.0	-0.116850	0.192621	0.080867	Uiso	?	C
C59	1.0	-0.334705	0.118128	0.199769	Uiso	?	C
C60	1.0	-0.068583	0.288319	0.082008	Uiso	?	C
C61	1.0	-0.241231	0.021697	0.196656	Uiso	?	C
C62	1.0	0.023737	0.191048	0.076926	Uiso	?	C
C63	1.0	-0.192979	0.117348	0.197806	Uiso	?	C
C64	1.0	0.072365	0.286962	0.078687	Uiso	?	C
C65	1.0	-0.097955	0.021999	0.197323	Uiso	?	C
C66	1.0	0.163589	0.189025	0.071843	Uiso	?	C
C67	1.0	-0.049678	0.117687	0.198597	Uiso	?	C
C68	1.0	0.213334	0.285545	0.075449	Uiso	?	C
C69	1.0	0.047545	0.024049	0.202612	Uiso	?	C
C70	1.0	0.305777	0.188532	0.070016	Uiso	?	C
C71	1.0	0.095726	0.119760	0.203617	Uiso	?	C
C72	1.0	0.355957	0.285375	0.075037	Uiso	?	C
C73	1.0	0.194978	0.027436	0.212044	Uiso	?	C

C74	1.0	0.451456	0.190255	0.075377	Uiso	? C
C75	1.0	0.243502	0.123977	0.212840	Uiso	? C
C76	1.0	0.500781	0.286772	0.078815	Uiso	? C
C77	1.0	0.339486	0.027665	0.212473	Uiso	? C
C78	1.0	0.597185	0.192464	0.080826	Uiso	? C
C79	1.0	0.387842	0.124017	0.212902	Uiso	? C
C80	1.0	0.645940	0.288515	0.082884	Uiso	? C
C81	1.0	0.477711	0.024797	0.204476	Uiso	? C
C82	1.0	0.741109	0.193232	0.082530	Uiso	? C
C83	1.0	0.525519	0.120210	0.204729	Uiso	? C
C84	1.0	0.789511	0.289032	0.083876	Uiso	? C
C85	1.0	0.618756	0.166542	0.201597	Uiso	? C
C86	1.0	-0.115308	0.336617	0.083638	Uiso	? C
C87	1.0	-0.334238	0.261349	0.200525	Uiso	? C
C88	1.0	-0.067511	0.432021	0.083840	Uiso	? C
C89	1.0	-0.240074	0.165356	0.198749	Uiso	? C
C90	1.0	0.026088	0.335523	0.080999	Uiso	? C
C91	1.0	-0.192342	0.260656	0.198927	Uiso	? C
C92	1.0	0.074053	0.431024	0.081536	Uiso	? C
C93	1.0	-0.097520	0.165161	0.198213	Uiso	? C
C94	1.0	0.167158	0.334208	0.077964	Uiso	? C
C95	1.0	-0.049627	0.260580	0.198690	Uiso	? C
C96	1.0	0.215529	0.429961	0.079245	Uiso	? C
C97	1.0	0.046645	0.166157	0.200605	Uiso	? C
C98	1.0	0.308965	0.333473	0.076199	Uiso	? C
C99	1.0	0.094078	0.261274	0.200226	Uiso	? C

C100	1.0	0.357717	0.429475	0.078195	Uiso	? C
C101	1.0	0.192885	0.169040	0.207078	Uiso	? C
C102	1.0	0.452817	0.334122	0.078019	Uiso	? C
C103	1.0	0.239126	0.263126	0.204097	Uiso	? C
C104	1.0	0.501365	0.430025	0.079655	Uiso	? C
C105	1.0	0.339823	0.172175	0.212990	Uiso	? C
C106	1.0	0.597782	0.335736	0.082025	Uiso	? C
C107	1.0	0.383884	0.264566	0.206894	Uiso	? C
C108	1.0	0.645932	0.431364	0.082775	Uiso	? C
C109	1.0	0.479677	0.169232	0.207470	Uiso	? C
C110	1.0	0.742090	0.336840	0.084413	Uiso	? C
C111	1.0	0.525122	0.263046	0.204015	Uiso	? C
C112	1.0	0.789995	0.432324	0.084698	Uiso	? C
C113	1.0	0.618176	0.309028	0.200627	Uiso	? C
C114	1.0	-0.114899	0.479892	0.084277	Uiso	? C
C115	1.0	-0.335879	0.403041	0.197769	Uiso	? C
C116	1.0	-0.068113	0.574530	0.082623	Uiso	? C
C117	1.0	-0.239892	0.308356	0.199097	Uiso	? C
C118	1.0	0.027030	0.479158	0.082591	Uiso	? C
C119	1.0	-0.192850	0.403179	0.198033	Uiso	? C
C120	1.0	0.074009	0.573947	0.081377	Uiso	? C
C121	1.0	-0.097269	0.308197	0.198658	Uiso	? C
C122	1.0	0.168600	0.478143	0.080432	Uiso	? C
C123	1.0	-0.049718	0.403380	0.198505	Uiso	? C
C124	1.0	0.216047	0.573293	0.080054	Uiso	? C
C125	1.0	0.045801	0.308350	0.199024	Uiso	? C

C126	1.0	0.310522	0.477425	0.078863	Uiso	?	C
C127	1.0	0.093227	0.403462	0.198624	Uiso	?	C
C128	1.0	0.358324	0.572792	0.079082	Uiso	?	C
C129	1.0	0.189679	0.309171	0.200881	Uiso	?	C
C130	1.0	0.453422	0.477454	0.078985	Uiso	?	C
C131	1.0	0.236275	0.403609	0.198943	Uiso	?	C
C132	1.0	0.501172	0.572758	0.079139	Uiso	?	C
C133	1.0	0.334181	0.310586	0.203663	Uiso	?	C
C134	1.0	0.597492	0.478362	0.081215	Uiso	?	C
C135	1.0	0.379393	0.403790	0.199423	Uiso	?	C
C136	1.0	0.644890	0.573456	0.080647	Uiso	?	C
C137	1.0	0.477056	0.310429	0.203367	Uiso	?	C
C138	1.0	0.741881	0.479581	0.083838	Uiso	?	C
C139	1.0	0.521801	0.403417	0.198707	Uiso	?	C
C140	1.0	0.788872	0.574364	0.082470	Uiso	?	C
C141	1.0	0.615717	0.450045	0.196415	Uiso	?	C
C142	1.0	-0.116291	0.621761	0.081635	Uiso	?	C
C143	1.0	-0.338183	0.544141	0.193690	Uiso	?	C
C144	1.0	-0.070436	0.715668	0.078461	Uiso	?	C
C145	1.0	-0.241172	0.450276	0.196824	Uiso	?	C
C146	1.0	0.026111	0.621444	0.080828	Uiso	?	C
C147	1.0	-0.194393	0.544888	0.195248	Uiso	?	C
C148	1.0	0.071662	0.715080	0.077231	Uiso	?	C
C149	1.0	-0.097642	0.450790	0.197983	Uiso	?	C
C150	1.0	0.168379	0.620943	0.079943	Uiso	?	C
C151	1.0	-0.050245	0.545879	0.197556	Uiso	?	C

C152	1.0	0.214768	0.715257	0.077695	Uiso	? C
C153	1.0	0.045484	0.450990	0.198442	Uiso	? C
C154	1.0	0.310757	0.620515	0.079220	Uiso	? C
C155	1.0	0.093126	0.546230	0.198415	Uiso	? C
C156	1.0	0.357946	0.715415	0.078408	Uiso	? C
C157	1.0	0.188211	0.450915	0.198232	Uiso	? C
C158	1.0	0.453374	0.620295	0.078832	Uiso	? C
C159	1.0	0.235550	0.545927	0.197664	Uiso	? C
C160	1.0	0.500917	0.715507	0.078603	Uiso	? C
C161	1.0	0.330887	0.450779	0.197985	Uiso	? C
C162	1.0	0.596624	0.620560	0.079421	Uiso	? C
C163	1.0	0.377532	0.545280	0.196204	Uiso	? C
C164	1.0	0.643932	0.715589	0.078768	Uiso	? C
C165	1.0	0.473403	0.450479	0.197386	Uiso	? C
C166	1.0	0.740337	0.621273	0.080883	Uiso	? C
C167	1.0	0.519396	0.544485	0.194534	Uiso	? C
C168	1.0	0.786980	0.715757	0.079045	Uiso	? C
C169	1.0	0.613732	0.591465	0.192911	Uiso	? C
C170	1.0	-0.118704	0.762731	0.077431	Uiso	? C
C171	1.0	-0.339063	0.686439	0.192022	Uiso	? C
C172	1.0	-0.072849	0.856707	0.074439	Uiso	? C
C173	1.0	-0.243001	0.591741	0.193452	Uiso	? C
C174	1.0	0.023100	0.762010	0.075521	Uiso	? C
C175	1.0	-0.195553	0.686878	0.192934	Uiso	? C
C176	1.0	0.067249	0.854520	0.069879	Uiso	? C
C177	1.0	-0.098569	0.592959	0.196213	Uiso	? C

C178	1.0	0.165749	0.761885	0.075313	Uiso	?	C
C179	1.0	-0.050720	0.688385	0.196552	Uiso	?	C
C180	1.0	0.210590	0.854738	0.070316	Uiso	?	C
C181	1.0	0.045454	0.593851	0.198332	Uiso	?	C
C182	1.0	0.309589	0.762488	0.077134	Uiso	?	C
C183	1.0	0.093651	0.689457	0.199385	Uiso	?	C
C184	1.0	0.356217	0.856972	0.075239	Uiso	?	C
C185	1.0	0.188241	0.593788	0.198260	Uiso	?	C
C186	1.0	0.453136	0.762988	0.078308	Uiso	?	C
C187	1.0	0.236382	0.689371	0.199108	Uiso	?	C
C188	1.0	0.500696	0.858212	0.078157	Uiso	?	C
C189	1.0	0.330183	0.593131	0.196706	Uiso	?	C
C190	1.0	0.596123	0.763117	0.078506	Uiso	?	C
C191	1.0	0.377889	0.688435	0.196798	Uiso	?	C
C192	1.0	0.643799	0.858373	0.078576	Uiso	?	C
C193	1.0	0.471827	0.592230	0.194633	Uiso	?	C
C194	1.0	0.738896	0.763053	0.078333	Uiso	?	C
C195	1.0	0.519134	0.687263	0.194004	Uiso	?	C
C196	1.0	0.786063	0.857987	0.077439	Uiso	?	C
Eu1	1.0	0.250987	0.050530	0.138792	Uiso	?	Eu

2. Eu-BG(AB).cif

```
#======================================================================
# CRYSTAL DATA
#----------------------------------------------------------------------
data_VESTA_phase_1

_chemical_name_common                  'POSCAR_AB_Eu                    '
_cell_length_a                         17.254942
_cell_length_b                         17.258297
_cell_length_c                         27.482088
_cell_angle_alpha                      95.020447
_cell_angle_beta                       93.945381
_cell_angle_gamma                      120.041382
_cell_volume                           6996.903923
_space_group_name_H-M_alt              'P 1'
_space_group_IT_number                 1

loop_
_space_group_symop_operation_xyz
   'x, y, z'

loop_
   _atom_site_label
   _atom_site_occupancy
   _atom_site_fract_x
```

_atom_site_fract_y

_atom_site_fract_z

_atom_site_adp_type

_atom_site_U_iso_or_equiv

_atom_site_type_symbol

C1	1.0	-0.140980	0.839438	0.216828	Uiso	?	C
C2	1.0	0.932428	0.935945	0.086161	Uiso	?	C
C3	1.0	-0.093264	-0.065171	0.217220	Uiso	?	C
C4	1.0	0.027060	-0.016970	0.084015	Uiso	?	C
C5	1.0	0.001435	0.838985	0.214523	Uiso	?	C
C6	1.0	0.073961	0.934606	0.081186	Uiso	?	C
C7	1.0	0.049442	-0.065390	0.216030	Uiso	?	C
C8	1.0	0.167982	-0.018732	0.077239	Uiso	?	C
C9	1.0	0.144700	0.839445	0.215816	Uiso	?	C
C10	1.0	0.214423	0.932341	0.073075	Uiso	?	C
C11	1.0	0.193092	-0.064392	0.220133	Uiso	?	C
C12	1.0	0.308414	-0.021352	0.067802	Uiso	?	C
C13	1.0	0.288705	-0.159522	0.221641	Uiso	?	C
C14	1.0	0.356725	0.930942	0.069749	Uiso	?	C
C15	1.0	0.337388	-0.062970	0.229430	Uiso	?	C
C16	1.0	0.454373	-0.019405	0.075231	Uiso	?	C
C17	1.0	0.432403	-0.158839	0.224242	Uiso	?	C
C18	1.0	0.502692	0.934383	0.080783	Uiso	?	C
C19	1.0	0.481196	-0.062477	0.229803	Uiso	?	C
C20	1.0	0.598922	-0.016803	0.085833	Uiso	?	C
C21	1.0	0.574742	-0.159274	0.222424	Uiso	?	C

C22	1.0	0.646907	0.936142	0.087674	Uiso	? C
C23	1.0	0.622794	-0.063488	0.224606	Uiso	? C
C24	1.0	0.742594	-0.015750	0.089278	Uiso	? C
C25	1.0	0.716879	0.840120	0.219794	Uiso	? C
C26	1.0	0.790125	-0.063463	0.088624	Uiso	? C
C27	1.0	0.764596	-0.064433	0.220364	Uiso	? C
C28	1.0	0.885187	-0.016014	0.087892	Uiso	? C
C29	1.0	-0.140541	-0.017150	0.218999	Uiso	? C
C30	1.0	0.932885	0.079342	0.088431	Uiso	? C
C31	1.0	-0.092842	0.078210	0.219546	Uiso	? C
C32	1.0	0.027738	0.126615	0.087039	Uiso	? C
C33	1.0	0.001877	-0.017595	0.216880	Uiso	? C
C34	1.0	0.074695	0.078371	0.084536	Uiso	? C
C35	1.0	0.049758	0.077944	0.218180	Uiso	? C
C36	1.0	0.169536	0.125659	0.082801	Uiso	? C
C37	1.0	0.145291	-0.017049	0.218864	Uiso	? C
C38	1.0	0.215893	0.077133	0.078591	Uiso	? C
C39	1.0	0.193424	0.078983	0.221601	Uiso	? C
C40	1.0	0.311140	0.124664	0.077634	Uiso	? C
C41	1.0	0.289631	-0.015309	0.227965	Uiso	? C
C42	1.0	0.357347	0.076055	0.072145	Uiso	? C
C43	1.0	0.337973	0.081589	0.232175	Uiso	? C
C44	1.0	0.454948	0.125156	0.078014	Uiso	? C
C45	1.0	0.434989	-0.013868	0.235306	Uiso	? C
C46	1.0	0.502709	0.077496	0.079461	Uiso	? C
C47	1.0	0.483922	0.083538	0.239623	Uiso	? C

C48	1.0	0.599247	0.126570	0.087297	Uiso	? C
C49	1.0	0.576439	-0.014959	0.228833	Uiso	? C
C50	1.0	0.647050	0.079229	0.088575	Uiso	? C
C51	1.0	0.624352	0.080906	0.230187	Uiso	? C
C52	1.0	0.742901	0.127578	0.091419	Uiso	? C
C53	1.0	0.717633	-0.016193	0.222865	Uiso	? C
C54	1.0	0.790471	0.079788	0.090574	Uiso	? C
C55	1.0	0.765273	0.079151	0.223400	Uiso	? C
C56	1.0	0.885610	0.127363	0.090221	Uiso	? C
C57	1.0	-0.140086	0.126248	0.221275	Uiso	? C
C58	1.0	0.933332	0.222756	0.090621	Uiso	? C
C59	1.0	-0.092548	0.221437	0.220948	Uiso	? C
C60	1.0	0.028208	0.270025	0.089131	Uiso	? C
C61	1.0	0.002228	0.125660	0.218834	Uiso	? C
C62	1.0	0.075468	0.222064	0.087621	Uiso	? C
C63	1.0	0.049878	0.221020	0.219158	Uiso	? C
C64	1.0	0.170486	0.269490	0.086786	Uiso	? C
C65	1.0	0.145444	0.126044	0.219777	Uiso	? C
C66	1.0	0.217598	0.221445	0.084982	Uiso	? C
C67	1.0	0.193035	0.221416	0.220501	Uiso	? C
C68	1.0	0.313004	0.269036	0.085506	Uiso	? C
C69	1.0	0.289653	0.127795	0.226621	Uiso	? C
C70	1.0	0.359943	0.221023	0.083176	Uiso	? C
C71	1.0	0.337128	0.223029	0.226123	Uiso	? C
C72	1.0	0.456203	0.269389	0.086345	Uiso	? C
C73	1.0	0.435616	0.131243	0.237651	Uiso	? C

C74	1.0	0.503639	0.221708	0.085771	Uiso	?	C
C75	1.0	0.481874	0.224899	0.232545	Uiso	?	C
C76	1.0	0.599857	0.270329	0.091085	Uiso	?	C
C77	1.0	0.577913	0.129837	0.234354	Uiso	?	C
C78	1.0	0.647641	0.222732	0.091609	Uiso	?	C
C79	1.0	0.624584	0.224315	0.230891	Uiso	?	C
C80	1.0	0.743413	0.270970	0.093814	Uiso	?	C
C81	1.0	0.718374	0.127571	0.226239	Uiso	?	C
C82	1.0	0.790913	0.223202	0.092930	Uiso	?	C
C83	1.0	0.765735	0.222538	0.225116	Uiso	?	C
C84	1.0	0.886079	0.270740	0.092134	Uiso	?	C
C85	1.0	-0.139855	0.269339	0.222045	Uiso	?	C
C86	1.0	0.933559	0.365847	0.091459	Uiso	?	C
C87	1.0	-0.092749	0.364029	0.220089	Uiso	?	C
C88	1.0	0.028234	0.412945	0.089306	Uiso	?	C
C89	1.0	0.002319	0.268669	0.219480	Uiso	?	C
C90	1.0	0.075706	0.365149	0.088678	Uiso	?	C
C91	1.0	0.049683	0.363640	0.218475	Uiso	?	C
C92	1.0	0.170683	0.412492	0.087570	Uiso	?	C
C93	1.0	0.145183	0.268692	0.219371	Uiso	?	C
C94	1.0	0.218196	0.364812	0.087389	Uiso	?	C
C95	1.0	0.192642	0.363778	0.218984	Uiso	?	C
C96	1.0	0.313630	0.412473	0.087652	Uiso	?	C
C97	1.0	0.288845	0.269489	0.222549	Uiso	?	C
C98	1.0	0.361178	0.364894	0.087724	Uiso	?	C
C99	1.0	0.336234	0.364520	0.221573	Uiso	?	C

C100	1.0	0.456836	0.412880	0.089202	Uiso	? C
C101	1.0	0.433132	0.271238	0.228049	Uiso	? C
C102	1.0	0.504542	0.365279	0.089797	Uiso	? C
C103	1.0	0.479979	0.365357	0.224944	Uiso	? C
C104	1.0	0.600331	0.413530	0.092036	Uiso	? C
C105	1.0	0.576835	0.272057	0.230417	Uiso	? C
C106	1.0	0.648102	0.366122	0.093260	Uiso	? C
C107	1.0	0.623170	0.365810	0.226012	Uiso	? C
C108	1.0	0.743679	0.414055	0.093863	Uiso	? C
C109	1.0	0.718486	0.270544	0.226431	Uiso	? C
C110	1.0	0.791274	0.366455	0.093971	Uiso	? C
C111	1.0	0.765267	0.364950	0.223282	Uiso	? C
C112	1.0	0.886119	0.413691	0.092237	Uiso	? C
C113	1.0	-0.140349	0.411696	0.220168	Uiso	? C
C114	1.0	0.933288	0.508375	0.090035	Uiso	? C
C115	1.0	-0.093386	0.506138	0.217365	Uiso	? C
C116	1.0	0.027797	0.555275	0.087302	Uiso	? C
C117	1.0	0.001954	0.411144	0.218078	Uiso	? C
C118	1.0	0.075401	0.507617	0.087404	Uiso	? C
C119	1.0	0.049145	0.505840	0.216056	Uiso	? C
C120	1.0	0.170226	0.554758	0.085651	Uiso	? C
C121	1.0	0.144818	0.411121	0.217972	Uiso	? C
C122	1.0	0.218005	0.507395	0.086529	Uiso	? C
C123	1.0	0.192219	0.506117	0.217006	Uiso	? C
C124	1.0	0.313340	0.555037	0.086371	Uiso	? C
C125	1.0	0.288176	0.411596	0.219699	Uiso	? C

C126	1.0	0.361253	0.507735	0.087600	Uiso	? C
C127	1.0	0.335640	0.506645	0.219085	Uiso	? C
C128	1.0	0.456699	0.555535	0.088350	Uiso	? C
C129	1.0	0.431715	0.412332	0.222422	Uiso	? C
C130	1.0	0.504592	0.508171	0.089631	Uiso	? C
C131	1.0	0.479001	0.507145	0.221061	Uiso	? C
C132	1.0	0.600122	0.556064	0.090418	Uiso	? C
C133	1.0	0.575215	0.412998	0.224385	Uiso	? C
C134	1.0	0.648004	0.508806	0.091717	Uiso	? C
C135	1.0	0.622110	0.507424	0.221764	Uiso	? C
C136	1.0	0.743191	0.556331	0.091342	Uiso	? C
C137	1.0	0.717731	0.412584	0.223198	Uiso	? C
C138	1.0	0.790981	0.508882	0.092159	Uiso	? C
C139	1.0	0.764533	0.506895	0.220096	Uiso	? C
C140	1.0	0.885715	0.556031	0.090025	Uiso	? C
C141	1.0	-0.140956	0.553798	0.217370	Uiso	? C
C142	1.0	0.932680	0.650471	0.087220	Uiso	? C
C143	1.0	-0.093777	0.648490	0.215191	Uiso	? C
C144	1.0	0.027064	0.697216	0.084110	Uiso	? C
C145	1.0	0.001351	0.553286	0.215234	Uiso	? C
C146	1.0	0.074602	0.649582	0.084189	Uiso	? C
C147	1.0	0.048658	0.648116	0.213534	Uiso	? C
C148	1.0	0.169166	0.696361	0.081375	Uiso	? C
C149	1.0	0.144336	0.553371	0.215689	Uiso	? C
C150	1.0	0.217121	0.649176	0.083010	Uiso	? C
C151	1.0	0.192010	0.648644	0.215353	Uiso	? C

C152	1.0	0.312358	0.696818	0.082453	Uiso	? C
C153	1.0	0.287749	0.554012	0.217798	Uiso	? C
C154	1.0	0.360622	0.649846	0.084994	Uiso	? C
C155	1.0	0.335510	0.649299	0.218214	Uiso	? C
C156	1.0	0.456106	0.697659	0.085848	Uiso	? C
C157	1.0	0.431088	0.554447	0.219840	Uiso	? C
C158	1.0	0.504164	0.650584	0.087738	Uiso	? C
C159	1.0	0.478722	0.649711	0.219787	Uiso	? C
C160	1.0	0.599703	0.698408	0.088468	Uiso	? C
C161	1.0	0.574337	0.554790	0.220907	Uiso	? C
C162	1.0	0.647529	0.651068	0.089476	Uiso	? C
C163	1.0	0.621674	0.649695	0.219878	Uiso	? C
C164	1.0	0.742665	0.698551	0.088968	Uiso	? C
C165	1.0	0.716937	0.554565	0.220017	Uiso	? C
C166	1.0	0.790388	0.651035	0.089339	Uiso	? C
C167	1.0	0.764117	0.649246	0.218140	Uiso	? C
C168	1.0	0.885088	0.698144	0.087314	Uiso	? C
C169	1.0	-0.141207	0.696347	0.215999	Uiso	? C
C170	1.0	0.932199	0.792842	0.085380	Uiso	? C
C171	1.0	-0.093729	0.791453	0.215322	Uiso	? C
C172	1.0	0.026605	0.839637	0.082299	Uiso	? C
C173	1.0	0.001067	0.695720	0.213454	Uiso	? C
C174	1.0	0.073850	0.791625	0.080975	Uiso	? C
C175	1.0	0.048927	0.791206	0.213614	Uiso	? C
C176	1.0	0.167752	0.837854	0.076516	Uiso	? C
C177	1.0	0.144239	0.696056	0.214134	Uiso	? C

C178	1.0	0.215511	0.790120	0.076984	Uiso	? C
C179	1.0	0.192486	0.791852	0.216321	Uiso	? C
C180	1.0	0.310474	0.837281	0.074850	Uiso	? C
C181	1.0	0.287804	0.696899	0.217607	Uiso	? C
C182	1.0	0.359214	0.790935	0.079344	Uiso	? C
C183	1.0	0.336143	0.792793	0.221068	Uiso	? C
C184	1.0	0.455219	0.839145	0.081275	Uiso	? C
C185	1.0	0.431171	0.697289	0.219704	Uiso	? C
C186	1.0	0.503503	0.792691	0.084873	Uiso	? C
C187	1.0	0.479341	0.793145	0.221912	Uiso	? C
C188	1.0	0.599318	0.840769	0.086947	Uiso	? C
C189	1.0	0.574159	0.697369	0.220052	Uiso	? C
C190	1.0	0.647172	0.793497	0.088092	Uiso	? C
C191	1.0	0.621861	0.792686	0.220630	Uiso	? C
C192	1.0	0.742479	0.841178	0.088309	Uiso	? C
C193	1.0	0.716650	0.697044	0.218770	Uiso	? C
C194	1.0	0.790039	0.793530	0.087982	Uiso	? C
C195	1.0	0.764144	0.792165	0.218304	Uiso	? C
C196	1.0	0.884899	0.840756	0.086492	Uiso	? C
Eu1	1.0	0.396143	0.030984	0.153715	Uiso	? Eu

3. Eu-NPBG(AA).cif

```
#======================================================================
# CRYSTAL DATA
#----------------------------------------------------------------------
data_VESTA_phase_1

_chemical_name_common                  'AA-bigraph-Pt                    '
_cell_length_a                         14.740000
_cell_length_b                         14.740000
_cell_length_c                         30.000000
_cell_angle_alpha                      90.000000
_cell_angle_beta                       90.000000
_cell_angle_gamma                      120.000000
_cell_volume                           5644.777655
_space_group_name_H-M_alt              'P 1'
_space_group_IT_number                 1

loop_
_space_group_symop_operation_xyz
   'x, y, z'

loop_
   _atom_site_label
   _atom_site_occupancy
   _atom_site_fract_x
```

_atom_site_fract_y

_atom_site_fract_z

_atom_site_adp_type

_atom_site_U_iso_or_equiv

_atom_site_type_symbol

C1 1.0 0.000053 0.000483 0.325966 Uiso ? C

C2 1.0 0.165613 0.000244 0.331930 Uiso ? C

C3 1.0 0.333361 0.000208 0.331694 Uiso ? C

C4 1.0 0.499315 0.000462 0.325603 Uiso ? C

C5 1.0 0.665635 -0.000394 0.323642 Uiso ? C

C6 1.0 0.832971 -0.000312 0.323781 Uiso ? C

C7 1.0 -0.000118 0.166721 0.329175 Uiso ? C

C8 1.0 0.163382 0.162816 0.346013 Uiso ? C

C9 1.0 0.319280 0.139505 0.368870 Uiso ? C

C10 1.0 0.498211 0.162567 0.345892 Uiso ? C

C11 1.0 0.665914 0.166570 0.329434 Uiso ? C

C12 1.0 0.832970 0.166846 0.323804 Uiso ? C

C13 1.0 0.000051 0.333131 0.325970 Uiso ? C

C14 1.0 0.163399 0.334151 0.345990 Uiso ? C

C15 1.0 0.670194 0.333960 0.346166 Uiso ? C

C16 1.0 0.832173 0.333097 0.326281 Uiso ? C

C17 1.0 -0.000975 0.499885 0.324413 Uiso ? C

C18 1.0 0.165617 0.498938 0.331897 Uiso ? C

C19 1.0 0.319293 0.513382 0.368836 Uiso ? C

C20 1.0 0.680540 0.507061 0.366640 Uiso ? C

C21 1.0 0.831178 0.498678 0.332517 Uiso ? C

C22	1.0	0.997941	0.665751	0.325406	Uiso	? C
C23	1.0	0.166160	0.665900	0.324901	Uiso	? C
C24	1.0	0.333358	0.666740	0.331649	Uiso	? C
C25	1.0	0.498207	0.669238	0.345831	Uiso	? C
C26	1.0	0.670178	0.669801	0.346124	Uiso	? C
C27	1.0	0.831170	0.666051	0.332505	Uiso	? C
C28	1.0	-0.000973	0.832703	0.324421	Uiso	? C
C29	1.0	0.166162	0.833836	0.324932	Uiso	? C
C30	1.0	0.332343	0.832970	0.323635	Uiso	? C
C31	1.0	0.499312	0.832452	0.325569	Uiso	? C
C32	1.0	0.665911	0.832911	0.329368	Uiso	? C
C33	1.0	0.832169	0.832632	0.326236	Uiso	? C
C34	1.0	0.000266	0.000650	0.458050	Uiso	? C
C35	1.0	0.165846	0.000426	0.452204	Uiso	? C
C36	1.0	0.333597	0.000389	0.452359	Uiso	? C
C37	1.0	0.499565	0.000635	0.458255	Uiso	? C
C38	1.0	0.665877	-0.000214	0.460095	Uiso	? C
C39	1.0	0.833194	-0.000141	0.460031	Uiso	? C
C40	1.0	0.000115	0.166899	0.454830	Uiso	? C
C41	1.0	0.163648	0.163008	0.438120	Uiso	? C
C42	1.0	0.319422	0.139642	0.415210	Uiso	? C
C43	1.0	0.498406	0.162743	0.438075	Uiso	? C
C44	1.0	0.666151	0.166747	0.454394	Uiso	? C
C45	1.0	0.833199	0.167024	0.460056	Uiso	? C
C46	1.0	0.000280	0.333312	0.458071	Uiso	? C
C47	1.0	0.163650	0.334330	0.438105	Uiso	? C

C48	1.0	0.670433	0.334134	0.437685	Uiso	?	C
C49	1.0	0.832393	0.333277	0.457660	Uiso	?	C
C50	1.0	-0.000746	0.500066	0.459636	Uiso	?	C
C51	1.0	0.165857	0.499120	0.452189	Uiso	?	C
C52	1.0	0.319413	0.513468	0.415173	Uiso	?	C
C53	1.0	0.680926	0.507286	0.417244	Uiso	?	C
C54	1.0	0.831416	0.498861	0.451454	Uiso	?	C
C55	1.0	0.998168	0.665928	0.458647	Uiso	?	C
C56	1.0	0.166406	0.666081	0.459160	Uiso	?	C
C57	1.0	0.333598	0.666912	0.452321	Uiso	?	C
C58	1.0	0.498409	0.669380	0.438022	Uiso	?	C
C59	1.0	0.670416	0.669977	0.437653	Uiso	?	C
C60	1.0	0.831402	0.666228	0.451434	Uiso	?	C
C61	1.0	-0.000757	0.832871	0.459616	Uiso	?	C
C62	1.0	0.166402	0.834017	0.459160	Uiso	?	C
C63	1.0	0.332600	0.833153	0.460341	Uiso	?	C
C64	1.0	0.499564	0.832640	0.458233	Uiso	?	C
C65	1.0	0.666147	0.833097	0.454367	Uiso	?	C
C66	1.0	0.832382	0.832802	0.457625	Uiso	?	C
C67	1.0	0.055601	0.110703	0.331703	Uiso	?	C
C68	1.0	0.217830	0.108202	0.346034	Uiso	?	C
C69	1.0	0.389113	0.107928	0.345955	Uiso	?	C
C70	1.0	0.554105	0.110638	0.331597	Uiso	?	C
C71	1.0	0.721185	0.111358	0.324503	Uiso	?	C
C72	1.0	0.889266	0.111412	0.324439	Uiso	?	C
C73	1.0	0.055605	0.278461	0.331694	Uiso	?	C

C74	1.0	0.194750	0.264175	0.368908	Uiso	? C
C75	1.0	0.568186	0.263988	0.368655	Uiso	? C
C76	1.0	0.722096	0.278347	0.332307	Uiso	? C
C77	1.0	0.888204	0.277439	0.323904	Uiso	? C
C78	1.0	0.055387	0.444276	0.326168	Uiso	? C
C79	1.0	0.217844	0.443210	0.346003	Uiso	? C
C80	1.0	0.723042	0.443414	0.346426	Uiso	? C
C81	1.0	0.887162	0.444239	0.326590	Uiso	? C
C82	1.0	0.054319	0.610408	0.324528	Uiso	? C
C83	1.0	0.221546	0.610682	0.329434	Uiso	? C
C84	1.0	0.389118	0.614775	0.345905	Uiso	? C
C85	1.0	0.568189	0.637834	0.368616	Uiso	? C
C86	1.0	0.723022	0.613187	0.346406	Uiso	? C
C87	1.0	0.887194	0.610374	0.330108	Uiso	? C
C88	1.0	0.054321	0.777474	0.324549	Uiso	? C
C89	1.0	0.221713	0.777646	0.324041	Uiso	? C
C90	1.0	0.388086	0.776863	0.325622	Uiso	? C
C91	1.0	0.554103	0.777058	0.331532	Uiso	? C
C92	1.0	0.722086	0.777304	0.332243	Uiso	? C
C93	1.0	0.887157	0.776477	0.326574	Uiso	? C
C94	1.0	0.055389	0.944676	0.326180	Uiso	? C
C95	1.0	0.221549	0.944439	0.329478	Uiso	? C
C96	1.0	0.388091	0.944821	0.325649	Uiso	? C
C97	1.0	0.555040	0.944321	0.323511	Uiso	? C
C98	1.0	0.721188	0.943399	0.324466	Uiso	? C
C99	1.0	0.888208	0.944327	0.323873	Uiso	? C

C100	1.0	0.055832	0.110879	0.452362	Uiso	? C
C101	1.0	0.218066	0.108390	0.438113	Uiso	? C
C102	1.0	0.389323	0.108108	0.438086	Uiso	? C
C103	1.0	0.554348	0.110807	0.452279	Uiso	? C
C104	1.0	0.721426	0.111538	0.459287	Uiso	? C
C105	1.0	0.889479	0.111582	0.459444	Uiso	? C
C106	1.0	0.055839	0.278644	0.452367	Uiso	? C
C107	1.0	0.194965	0.264319	0.415182	Uiso	? C
C108	1.0	0.568302	0.264118	0.415256	Uiso	? C
C109	1.0	0.722324	0.278537	0.451558	Uiso	? C
C110	1.0	0.888425	0.277622	0.460050	Uiso	? C
C111	1.0	0.055620	0.444458	0.457900	Uiso	? C
C112	1.0	0.218071	0.443374	0.438085	Uiso	? C
C113	1.0	0.723327	0.443617	0.437463	Uiso	? C
C114	1.0	0.887387	0.444417	0.457404	Uiso	? C
C115	1.0	0.054556	0.610592	0.459539	Uiso	? C
C116	1.0	0.221793	0.610862	0.454616	Uiso	? C
C117	1.0	0.389322	0.614922	0.438034	Uiso	? C
C118	1.0	0.568315	0.637907	0.415209	Uiso	? C
C119	1.0	0.723304	0.613374	0.437441	Uiso	? C
C120	1.0	0.887421	0.610553	0.453896	Uiso	? C
C121	1.0	0.054551	0.777653	0.459526	Uiso	? C
C122	1.0	0.221962	0.777830	0.460013	Uiso	? C
C123	1.0	0.388342	0.777051	0.458285	Uiso	? C
C124	1.0	0.554343	0.777248	0.452243	Uiso	? C
C125	1.0	0.722312	0.777471	0.451528	Uiso	? C

C126	1.0	0.887377	0.776652	0.457375	Uiso	?	C
C127	1.0	0.055605	0.944846	0.457893	Uiso	?	C
C128	1.0	0.221791	0.944627	0.454630	Uiso	?	C
C129	1.0	0.388342	0.944997	0.458311	Uiso	?	C
C130	1.0	0.555292	0.944501	0.460260	Uiso	?	C
C131	1.0	0.721423	0.943579	0.459268	Uiso	?	C
C132	1.0	0.888413	0.944487	0.460013	Uiso	?	C
Eu1	1.0	0.481597	0.407457	0.392098	Uiso	?	Eu

4. Eu-NPBG(AB).cif

```
#======================================================================
# CRYSTAL DATA
#----------------------------------------------------------------------
data_VESTA_phase_1

_chemical_name_common                  'AB_bigraph                        '
_cell_length_a                         14.718637
_cell_length_b                         14.716187
_cell_length_c                         22.178364
_cell_angle_alpha                      90.382126
_cell_angle_beta                       90.062698
_cell_angle_gamma                      119.991257
_cell_volume                           4160.504036
_space_group_name_H-M_alt              'P 1'
_space_group_IT_number                 1

loop_
_space_group_symop_operation_xyz
   'x, y, z'

loop_
   _atom_site_label
   _atom_site_occupancy
   _atom_site_fract_x
```

_atom_site_fract_y

_atom_site_fract_z

_atom_site_adp_type

_atom_site_U_iso_or_equiv

_atom_site_type_symbol

C1	1.0	0.999842	0.999587	0.296380	Uiso	?	C
C2	1.0	0.167094	0.000322	0.291922	Uiso	?	C
C3	1.0	0.334407	0.000323	0.291897	Uiso	?	C
C4	1.0	0.500940	0.999589	0.296320	Uiso	?	C
C5	1.0	0.667137	0.999086	0.305050	Uiso	?	C
C6	1.0	0.833172	0.999087	0.305087	Uiso	?	C
C7	1.0	0.000527	0.166439	0.305269	Uiso	?	C
C8	1.0	0.167406	0.167232	0.306843	Uiso	?	C
C9	1.0	0.333823	0.166438	0.306357	Uiso	?	C
C10	1.0	0.501045	0.167257	0.306787	Uiso	?	C
C11	1.0	0.667125	0.166453	0.305185	Uiso	?	C
C12	1.0	0.833599	0.165992	0.305346	Uiso	?	C
C13	1.0	0.000513	0.332459	0.305284	Uiso	?	C
C14	1.0	0.167187	0.332981	0.323248	Uiso	?	C
C15	1.0	0.320301	0.308981	0.357606	Uiso	?	C
C16	1.0	0.490034	0.308965	0.357605	Uiso	?	C
C17	1.0	0.667054	0.333004	0.323202	Uiso	?	C
C18	1.0	0.833153	0.332464	0.305238	Uiso	?	C
C19	1.0	0.999815	0.498437	0.296406	Uiso	?	C
C20	1.0	0.167403	0.498581	0.306740	Uiso	?	C
C21	1.0	0.320368	0.510890	0.357486	Uiso	?	C

C22	1.0	0.691985	0.510945	0.357485	Uiso	? C
C23	1.0	0.832401	0.498591	0.306703	Uiso	? C
C24	1.0	0.998972	0.664865	0.291804	Uiso	? C
C25	1.0	0.167082	0.664860	0.291818	Uiso	? C
C26	1.0	0.333849	0.665780	0.306122	Uiso	? C
C27	1.0	0.490093	0.680615	0.357410	Uiso	? C
C28	1.0	0.691969	0.680652	0.357409	Uiso	? C
C29	1.0	0.833196	0.665810	0.306109	Uiso	? C
C30	1.0	0.998987	0.832211	0.291791	Uiso	? C
C31	1.0	0.166784	0.832392	0.287274	Uiso	? C
C32	1.0	0.334408	0.832198	0.291781	Uiso	? C
C33	1.0	0.501060	0.832225	0.306539	Uiso	? C
C34	1.0	0.667059	0.832835	0.322948	Uiso	? C
C35	1.0	0.832415	0.832243	0.306556	Uiso	? C
C36	1.0	0.002429	0.001975	0.484674	Uiso	? C
C37	1.0	0.169107	0.002478	0.475968	Uiso	? C
C38	1.0	0.335148	0.002484	0.475989	Uiso	? C
C39	1.0	0.501340	0.001982	0.484728	Uiso	? C
C40	1.0	0.667871	0.001244	0.489153	Uiso	? C
C41	1.0	0.835178	0.001242	0.489119	Uiso	? C
C42	1.0	0.003264	0.169343	0.489192	Uiso	? C
C43	1.0	0.169845	0.169312	0.474492	Uiso	? C
C44	1.0	0.335213	0.168737	0.458139	Uiso	? C
C45	1.0	0.501214	0.169351	0.474545	Uiso	? C
C46	1.0	0.667864	0.169371	0.489268	Uiso	? C
C47	1.0	0.835481	0.169169	0.493712	Uiso	? C

C48	1.0	0.003274	0.336682	0.489179	Uiso	? C
C49	1.0	0.169059	0.335744	0.474931	Uiso	? C
C50	1.0	0.310338	0.320934	0.423686	Uiso	? C
C51	1.0	0.512164	0.320970	0.423699	Uiso	? C
C52	1.0	0.668416	0.335800	0.474977	Uiso	? C
C53	1.0	0.835182	0.336704	0.489222	Uiso	? C
C54	1.0	0.002451	0.503124	0.484651	Uiso	? C
C55	1.0	0.169856	0.502965	0.474348	Uiso	? C
C56	1.0	0.310322	0.490636	0.423611	Uiso	? C
C57	1.0	0.681903	0.490688	0.423611	Uiso	? C
C58	1.0	0.834866	0.502995	0.474366	Uiso	? C
C59	1.0	0.001770	0.669112	0.475818	Uiso	? C
C60	1.0	0.169123	0.669098	0.475825	Uiso	? C
C61	1.0	0.335219	0.668557	0.457889	Uiso	? C
C62	1.0	0.512226	0.692573	0.423503	Uiso	? C
C63	1.0	0.681972	0.692576	0.423491	Uiso	? C
C64	1.0	0.835086	0.668588	0.457866	Uiso	? C
C65	1.0	0.001757	0.835129	0.475830	Uiso	? C
C66	1.0	0.168687	0.835577	0.475708	Uiso	? C
C67	1.0	0.335159	0.835118	0.475859	Uiso	? C
C68	1.0	0.501234	0.834306	0.474299	Uiso	? C
C69	1.0	0.668450	0.835113	0.474741	Uiso	? C
C70	1.0	0.834867	0.834326	0.474261	Uiso	? C
C71	1.0	0.055939	0.111107	0.301235	Uiso	? C
C72	1.0	0.222888	0.111392	0.300083	Uiso	? C
C73	1.0	0.389705	0.111401	0.300052	Uiso	? C

C74	1.0	0.556376	0.111121	0.301155	Uiso	?	C
C75	1.0	0.722623	0.110507	0.304373	Uiso	?	C
C76	1.0	0.889100	0.110502	0.304424	Uiso	?	C
C77	1.0	0.056967	0.277723	0.310073	Uiso	?	C
C78	1.0	0.220423	0.273974	0.326748	Uiso	?	C
C79	1.0	0.383480	0.265657	0.337519	Uiso	?	C
C80	1.0	0.554822	0.273993	0.326721	Uiso	?	C
C81	1.0	0.721976	0.277738	0.310002	Uiso	?	C
C82	1.0	0.889082	0.276955	0.304492	Uiso	?	C
C83	1.0	0.055918	0.443115	0.301214	Uiso	?	C
C84	1.0	0.220442	0.445304	0.326677	Uiso	?	C
C85	1.0	0.726153	0.445327	0.326644	Uiso	?	C
C86	1.0	0.888403	0.443120	0.301181	Uiso	?	C
C87	1.0	0.055344	0.609492	0.292188	Uiso	?	C
C88	1.0	0.222893	0.609749	0.299918	Uiso	?	C
C89	1.0	0.383537	0.616913	0.337317	Uiso	?	C
C90	1.0	0.734768	0.616957	0.337312	Uiso	?	C
C91	1.0	0.888086	0.609763	0.299893	Uiso	?	C
C92	1.0	0.054916	0.776472	0.288968	Uiso	?	C
C93	1.0	0.222744	0.776466	0.288971	Uiso	?	C
C94	1.0	0.389720	0.776580	0.299828	Uiso	?	C
C95	1.0	0.554845	0.779680	0.326454	Uiso	?	C
C96	1.0	0.726146	0.779698	0.326448	Uiso	?	C
C97	1.0	0.888098	0.776603	0.299833	Uiso	?	C
C98	1.0	0.055361	0.943963	0.292207	Uiso	?	C
C99	1.0	0.222749	0.944316	0.289053	Uiso	?	C

C100	1.0	0.389790	0.943960	0.292173	Uiso	? C
C101	1.0	0.556386	0.943589	0.301007	Uiso	? C
C102	1.0	0.721996	0.942754	0.309791	Uiso	? C
C103	1.0	0.888424	0.943597	0.301054	Uiso	? C
C104	1.0	0.113844	0.057962	0.479999	Uiso	? C
C105	1.0	0.280277	0.058809	0.471267	Uiso	? C
C106	1.0	0.445894	0.057984	0.480045	Uiso	? C
C107	1.0	0.612487	0.057611	0.488874	Uiso	? C
C108	1.0	0.779526	0.057252	0.491969	Uiso	? C
C109	1.0	0.946904	0.057597	0.488810	Uiso	? C
C110	1.0	0.114151	0.224947	0.481184	Uiso	? C
C111	1.0	0.276124	0.221868	0.454633	Uiso	? C
C112	1.0	0.447423	0.221903	0.454651	Uiso	? C
C113	1.0	0.612552	0.224995	0.481255	Uiso	? C
C114	1.0	0.779526	0.225096	0.492049	Uiso	? C
C115	1.0	0.947338	0.225080	0.492000	Uiso	? C
C116	1.0	0.114163	0.391786	0.481124	Uiso	? C
C117	1.0	0.267525	0.384620	0.443775	Uiso	? C
C118	1.0	0.618723	0.384670	0.443789	Uiso	? C
C119	1.0	0.779374	0.391821	0.481170	Uiso	? C
C120	1.0	0.946916	0.392065	0.488828	Uiso	? C
C121	1.0	0.113862	0.558440	0.479876	Uiso	? C
C122	1.0	0.276119	0.556240	0.454439	Uiso	? C
C123	1.0	0.781822	0.556270	0.454434	Uiso	? C
C124	1.0	0.946353	0.558455	0.479883	Uiso	? C
C125	1.0	0.113199	0.724611	0.476579	Uiso	? C

C126	1.0	0.280299	0.723828	0.471064	Uiso	?	C
C127	1.0	0.447449	0.727558	0.454384	Uiso	?	C
C128	1.0	0.618783	0.735886	0.443586	Uiso	?	C
C129	1.0	0.781844	0.727584	0.454360	Uiso	?	C
C130	1.0	0.945314	0.723849	0.471039	Uiso	?	C
C131	1.0	0.113187	0.891065	0.476642	Uiso	?	C
C132	1.0	0.279665	0.891064	0.476656	Uiso	?	C
C133	1.0	0.445909	0.890450	0.479901	Uiso	?	C
C134	1.0	0.612572	0.890160	0.481032	Uiso	?	C
C135	1.0	0.779387	0.890167	0.481006	Uiso	?	C
C136	1.0	0.946339	0.890456	0.479859	Uiso	?	C
Eu1	1.0	0.501125	0.500778	0.390517	Uiso	?	Eu

5. Eu-TBG.cif

```
#======================================================================
# CRYSTAL DATA
#----------------------------------------------------------------------
data_VESTA_phase_1

_chemical_name_common                  'Eu/TBG'
_cell_length_a                         16.931160
_cell_length_b                         16.904827
_cell_length_c                         22.296223
_cell_angle_alpha                      96.492416
_cell_angle_beta                       81.913322
_cell_angle_gamma                      118.406631
_cell_volume                           5549.578203
_space_group_name_H-M_alt              'P 1'
_space_group_IT_number                 1

loop_
_space_group_symop_operation_xyz
   'x, y, z'

loop_
   _atom_site_label
   _atom_site_occupancy
   _atom_site_fract_x
```

_atom_site_fract_y

_atom_site_fract_z

_atom_site_adp_type

_atom_site_U_iso_or_equiv

_atom_site_type_symbol

C1 1.0 0.017797 0.992793 0.965275 Uiso ? C

C2 1.0 0.113396 0.040079 0.963764 Uiso ? C

C3 1.0 0.160591 0.992374 0.970776 Uiso ? C

C4 1.0 0.255655 0.040372 0.973204 Uiso ? C

C5 1.0 0.300842 0.994201 0.987319 Uiso ? C

C6 1.0 0.395213 0.042493 0.992482 Uiso ? C

C7 1.0 0.440365 0.996405 0.006489 Uiso ? C

C8 1.0 0.534532 0.044340 0.012054 Uiso ? C

C9 1.0 0.582602 0.996075 0.013856 Uiso ? C

C10 1.0 0.678364 0.043725 0.012162 Uiso ? C

C11 1.0 0.727747 0.995265 0.999656 Uiso ? C

C12 1.0 0.823603 0.042894 0.992297 Uiso ? C

C13 1.0 0.872916 0.994508 0.977235 Uiso ? C

C14 1.0 0.968758 0.041713 0.970985 Uiso ? C

C15 1.0 0.015853 0.137399 0.975878 Uiso ? C

C16 1.0 0.111883 0.184518 0.967795 Uiso ? C

C17 1.0 0.161147 0.135549 0.961291 Uiso ? C

C18 1.0 0.257098 0.182799 0.959315 Uiso ? C

C19 1.0 0.304774 0.134840 0.965115 Uiso ? C

C20 1.0 0.399419 0.182968 0.970101 Uiso ? C

C21 1.0 0.444706 0.136749 0.984600 Uiso ? C

C22	1.0	0.537126	0.185608	0.996133	Uiso	? C
C23	1.0	0.582041	0.139463	0.010596	Uiso	? C
C24	1.0	0.675968	0.187994	0.019047	Uiso	? C
C25	1.0	0.724940	0.139181	0.017763	Uiso	? C
C26	1.0	0.820798	0.186692	0.014268	Uiso	? C
C27	1.0	0.870475	0.138303	0.000461	Uiso	? C
C28	1.0	0.966464	0.185792	0.991612	Uiso	? C
C29	1.0	0.013475	0.281207	0.998950	Uiso	? C
C30	1.0	0.109319	0.328463	0.988553	Uiso	? C
C31	1.0	0.158890	0.280238	0.971762	Uiso	? C
C32	1.0	0.254975	0.327271	0.963876	Uiso	? C
C33	1.0	0.304454	0.278441	0.958250	Uiso	? C
C34	1.0	0.399928	0.325781	0.960389	Uiso	? C
C35	1.0	0.447317	0.278124	0.967439	Uiso	? C
C36	1.0	0.540340	0.326737	0.978591	Uiso	? C
C37	1.0	0.584929	0.280755	0.994157	Uiso	? C
C38	1.0	0.675751	0.330181	0.009888	Uiso	? C
C39	1.0	0.722263	0.282928	0.021309	Uiso	? C
C40	1.0	0.818088	0.331547	0.025328	Uiso	? C
C41	1.0	0.867589	0.282480	0.019495	Uiso	? C
C42	1.0	0.963615	0.329654	0.014094	Uiso	? C
C43	1.0	0.010278	0.425257	0.019108	Uiso	? C
C44	1.0	0.106573	0.472306	0.012411	Uiso	? C
C45	1.0	0.156161	0.423945	0.996294	Uiso	? C
C46	1.0	0.252062	0.471132	0.986663	Uiso	? C
C47	1.0	0.301660	0.422905	0.970230	Uiso	? C

C48	1.0	0.397690	0.470093	0.966625	Uiso	?	C
C49	1.0	0.447068	0.421516	0.962321	Uiso	?	C
C50	1.0	0.541871	0.469048	0.967077	Uiso	?	C
C51	1.0	0.588364	0.421729	0.976854	Uiso	?	C
C52	1.0	0.680161	0.470406	0.990667	Uiso	?	C
C53	1.0	0.721578	0.424565	0.009732	Uiso	?	C
C54	1.0	0.811602	0.477383	0.036095	Uiso	?	C
C55	1.0	0.865185	0.426291	0.028363	Uiso	?	C
C56	1.0	0.960184	0.473886	0.026431	Uiso	?	C
C57	1.0	0.008863	0.570379	0.025103	Uiso	?	C
C58	1.0	0.104092	0.616940	0.022773	Uiso	?	C
C59	1.0	0.153574	0.567923	0.017615	Uiso	?	C
C60	1.0	0.249486	0.614807	0.011148	Uiso	?	C
C61	1.0	0.298885	0.566339	0.996150	Uiso	?	C
C62	1.0	0.394891	0.613851	0.988906	Uiso	?	C
C63	1.0	0.444381	0.565548	0.974417	Uiso	?	C
C64	1.0	0.540220	0.612890	0.971383	Uiso	?	C
C65	1.0	0.589047	0.564384	0.968415	Uiso	?	C
C66	1.0	0.683995	0.611980	0.973045	Uiso	?	C
C67	1.0	0.729190	0.565795	0.985723	Uiso	?	C
C68	1.0	0.823471	0.614532	0.993585	Uiso	?	C
C69	1.0	0.867560	0.571575	0.012986	Uiso	?	C
C70	1.0	0.961718	0.618011	0.015934	Uiso	?	C
C71	1.0	0.011963	0.711169	0.003677	Uiso	?	C
C72	1.0	0.106725	0.759379	0.007085	Uiso	?	C
C73	1.0	0.152871	0.712390	0.017638	Uiso	?	C

C74	1.0	0.248319	0.759516	0.016767	Uiso	? C
C75	1.0	0.296947	0.710657	0.014948	Uiso	? C
C76	1.0	0.392613	0.757623	0.009926	Uiso	? C
C77	1.0	0.441917	0.709042	0.997564	Uiso	? C
C78	1.0	0.537882	0.756697	0.990697	Uiso	? C
C79	1.0	0.587140	0.708502	0.977348	Uiso	? C
C80	1.0	0.683106	0.755893	0.972655	Uiso	? C
C81	1.0	0.731886	0.707451	0.970041	Uiso	? C
C82	1.0	0.826962	0.754714	0.970964	Uiso	? C
C83	1.0	0.873250	0.707964	0.982118	Uiso	? C
C84	1.0	0.967288	0.756478	0.987361	Uiso	? C
C85	1.0	0.016694	0.850290	0.977145	Uiso	? C
C86	1.0	0.111275	0.898538	0.980640	Uiso	? C
C87	1.0	0.156357	0.852760	0.995659	Uiso	? C
C88	1.0	0.250989	0.900946	0.998818	Uiso	? C
C89	1.0	0.296798	0.854190	0.010654	Uiso	? C
C90	1.0	0.391915	0.901646	0.011818	Uiso	? C
C91	1.0	0.439992	0.853295	0.012711	Uiso	? C
C92	1.0	0.535710	0.900649	0.009843	Uiso	? C
C93	1.0	0.585024	0.852166	0.998419	Uiso	? C
C94	1.0	0.680761	0.899845	0.991326	Uiso	? C
C95	1.0	0.730068	0.851508	0.977230	Uiso	? C
C96	1.0	0.825904	0.898791	0.971802	Uiso	? C
C97	1.0	0.874757	0.849925	0.967645	Uiso	? C
C98	1.0	0.970061	0.897402	0.967892	Uiso	? C
C99	1.0	0.742116	0.007591	0.170133	Uiso	? C

C100	1.0	0.570613	0.025892	0.172983	Uiso	? C
C101	1.0	0.373793	0.143858	0.099885	Uiso	? C
C102	1.0	0.659695	0.094959	0.185518	Uiso	? C
C103	1.0	0.245036	0.006717	0.158391	Uiso	? C
C104	1.0	0.337092	0.994416	0.157714	Uiso	? C
C105	1.0	0.430189	0.069770	0.155718	Uiso	? C
C106	1.0	0.516151	0.076422	0.170669	Uiso	? C
C107	1.0	0.579243	0.170616	0.180515	Uiso	? C
C108	1.0	0.264809	0.371767	0.095688	Uiso	? C
C109	1.0	0.061888	0.021418	0.177227	Uiso	? C
C110	1.0	0.152139	0.091790	0.170474	Uiso	? C
C111	1.0	0.234539	0.086779	0.152431	Uiso	? C
C112	1.0	0.308263	0.167185	0.128004	Uiso	? C
C113	1.0	0.437681	0.152741	0.135318	Uiso	? C
C114	1.0	0.493962	0.245924	0.157553	Uiso	? C
C115	1.0	0.074776	0.177086	0.189404	Uiso	? C
C116	1.0	0.159641	0.179542	0.176889	Uiso	? C
C117	1.0	0.238126	0.258549	0.165569	Uiso	? C
C118	1.0	0.318665	0.253769	0.144880	Uiso	? C
C119	1.0	0.404789	0.335233	0.144932	Uiso	? C
C120	1.0	0.834796	0.015954	0.170973	Uiso	? C
C121	1.0	0.915065	0.091121	0.190876	Uiso	? C
C122	1.0	0.002952	0.088731	0.189104	Uiso	? C
C123	1.0	0.741133	0.090618	0.186171	Uiso	? C
C124	1.0	0.823957	0.174036	0.196598	Uiso	? C
C125	1.0	0.908779	0.175250	0.199426	Uiso	? C

C126	1.0	0.987356	0.261201	0.201615	Uiso	? C
C127	1.0	0.662551	0.179396	0.191203	Uiso	? C
C128	1.0	0.740482	0.260596	0.195478	Uiso	? C
C129	1.0	0.822749	0.259365	0.199742	Uiso	? C
C130	1.0	0.902098	0.344232	0.201351	Uiso	? C
C131	1.0	0.985611	0.344924	0.201181	Uiso	? C
C132	1.0	0.573390	0.251291	0.175546	Uiso	? C
C133	1.0	0.652219	0.336650	0.178077	Uiso	? C
C134	1.0	0.736710	0.340871	0.187747	Uiso	? C
C135	1.0	0.816995	0.421228	0.185605	Uiso	? C
C136	1.0	0.899396	0.426831	0.195630	Uiso	? C
C137	1.0	0.980349	0.512787	0.192076	Uiso	? C
C138	1.0	0.484914	0.328549	0.152425	Uiso	? C
C139	1.0	0.563600	0.413183	0.160232	Uiso	? C
C140	1.0	0.645932	0.413495	0.166602	Uiso	? C
C141	1.0	0.728280	0.497955	0.154451	Uiso	? C
C142	1.0	0.820434	0.500774	0.163551	Uiso	? C
C143	1.0	0.892843	0.588773	0.182683	Uiso	? C
C144	1.0	0.977873	0.596161	0.187507	Uiso	? C
C145	1.0	0.071435	0.261399	0.194705	Uiso	? C
C146	1.0	0.150870	0.344281	0.184774	Uiso	? C
C147	1.0	0.232557	0.339951	0.162432	Uiso	? C
C148	1.0	0.319859	0.426045	0.147983	Uiso	? C
C149	1.0	0.066271	0.428655	0.194132	Uiso	? C
C150	1.0	0.149860	0.428086	0.185136	Uiso	? C
C151	1.0	0.231696	0.511809	0.175166	Uiso	? C

C152	1.0	0.063239	0.511804	0.191011	Uiso	? C
C153	1.0	0.142477	0.595211	0.183961	Uiso	? C
C154	1.0	0.053675	0.684568	0.181524	Uiso	? C
C155	1.0	0.403077	0.419417	0.150245	Uiso	? C
C156	1.0	0.481909	0.502981	0.155571	Uiso	? C
C157	1.0	0.561893	0.498217	0.158966	Uiso	? C
C158	1.0	0.641524	0.582205	0.163366	Uiso	? C
C159	1.0	0.791988	0.493445	0.103660	Uiso	? C
C160	1.0	0.804046	0.663759	0.170604	Uiso	? C
C161	1.0	0.879581	0.660509	0.183640	Uiso	? C
C162	1.0	0.316241	0.512036	0.163730	Uiso	? C
C163	1.0	0.397527	0.593869	0.161499	Uiso	? C
C164	1.0	0.480258	0.589329	0.157316	Uiso	? C
C165	1.0	0.560386	0.672316	0.156992	Uiso	? C
C166	1.0	0.641573	0.665999	0.159784	Uiso	? C
C167	1.0	0.726066	0.743575	0.150082	Uiso	? C
C168	1.0	0.812755	0.746408	0.156646	Uiso	? C
C169	1.0	0.905804	0.823514	0.152578	Uiso	? C
C170	1.0	0.996250	0.837164	0.167753	Uiso	? C
C171	1.0	0.228255	0.595295	0.177603	Uiso	? C
C172	1.0	0.311155	0.677860	0.171450	Uiso	? C
C173	1.0	0.396006	0.679410	0.165169	Uiso	? C
C174	1.0	0.475749	0.765719	0.161598	Uiso	? C
C175	1.0	0.559413	0.759387	0.155752	Uiso	? C
C176	1.0	0.644249	0.837562	0.147394	Uiso	? C
C177	1.0	0.720234	0.818671	0.132641	Uiso	? C

C178	1.0	0.860313	0.956679	0.137661	Uiso	? C
C179	1.0	0.891539	0.890867	0.127940	Uiso	? C
C180	1.0	0.723578	0.582932	0.165465	Uiso	? C
C181	1.0	0.137219	0.678129	0.180980	Uiso	? C
C182	1.0	0.220490	0.754857	0.175539	Uiso	? C
C183	1.0	0.307400	0.759638	0.171482	Uiso	? C
C184	1.0	0.380007	0.849314	0.166189	Uiso	? C
C185	1.0	0.473131	0.853432	0.162727	Uiso	? C
C186	1.0	0.563111	0.935448	0.164052	Uiso	? C
C187	1.0	0.651228	0.928116	0.158439	Uiso	? C
C188	1.0	0.056685	0.776005	0.174370	Uiso	? C
C189	1.0	0.147047	0.847716	0.172896	Uiso	? C
C190	1.0	0.227697	0.839383	0.172184	Uiso	? C
C191	1.0	0.322457	0.905451	0.165890	Uiso	? C
C192	1.0	0.807596	0.876546	0.110897	Uiso	? C
C193	1.0	0.064662	0.932333	0.171460	Uiso	? C
C194	1.0	0.156010	0.934970	0.169265	Uiso	? C
Eu1	1.0	0.347159	0.156595	0.231069	Uiso	? Eu
